# Integrative multi‐omic analysis reveals neurodevelopmental gene dysregulation in *CIC* ‐knockout and *IDH1* ‐mutant cells

**DOI:** 10.1002/path.5835

**Published:** 2021-12-22

**Authors:** Stephen D Lee, Jungeun Song, Veronique G LeBlanc, Marco A Marra

**Affiliations:** ^1^ Canada's Michael Smith Genome Sciences Centre, BC Cancer Vancouver Canada; ^2^ Department of Medical Genetics University of British Columbia Vancouver Canada

**Keywords:** capicua transcriptional repressor, neomorphic *IDH* mutation, epigenomics, transcriptomics

## Abstract

Capicua (CIC)'s transcriptional repressor function is implicated in neurodevelopment and in oligodendroglioma (ODG) aetiology. However, CIC's role in these contexts remains obscure, primarily from our currently limited knowledge regarding its biological functions. Moreover, *CIC* mutations in ODG invariably co‐occur with a neomorphic *IDH1/2* mutation, yet the functional relationship between these two genetic events is unknown. Here, we analysed models derived from an E6/E7/hTERT‐immortalized (i.e. p53‐ and RB‐deficient) normal human astrocyte cell line. To examine the consequences of CIC loss, we compared transcriptomic and epigenomic profiles between CIC wild‐type and knockout cell lines, with and without mutant IDH1 expression. Our analyses revealed dysregulation of neurodevelopmental genes in association with CIC loss. CIC ChIP‐seq was also performed to expand upon the currently limited ensemble of known CIC target genes. Among the newly identified direct CIC target genes were *EPHA2* and *ID1*, whose functions are linked to neurodevelopment and the tumourigenicity of *in vivo* glioma tumour models. *NFIA*, a known mediator of gliogenesis, was discovered to be uniquely overexpressed in *CIC*‐knockout cells expressing mutant IDH1‐R132H protein. These results identify neurodevelopment and specific genes within this context as candidate targets through which *CIC* alterations may contribute to the progression of *IDH*‐mutant gliomas. © 2021 The Authors. *The Journal of Pathology* published by John Wiley & Sons, Ltd on behalf of The Pathological Society of Great Britain and Ireland.

## Introduction

Capicua (CIC) functions downstream of receptor tyrosine kinase (RTK) signalling through a mechanism called default repression: in the absence of RTK signals, CIC maintains its transcriptional repressor activity, whereas induction of RTK signalling results in inactivation of CIC and subsequent de‐repression of its target genes [1, 2]. To date, CIC has been implicated in a broad range of physiological processes, including lung alveolarization [3], bile acid homeostasis [4], and T‐cell development [5, 6]. Furthermore, CIC activity appears to be important in neurodevelopment, as its dysfunction has been linked to a spectrum of neuro‐behavioural syndromes [7], neurodegeneration [8], and altered lineage specification of neural stem cells (NSCs) [9, 10, 11].

Among brain tumours, *CIC* is mutated almost exclusively and at high frequency (~50–80%) in oligodendroglioma (ODG) [12, 13, 14, 15]. Defining molecular characteristics of ODG include *IDH1/2* mutation and single copy deletion of chromosome arms 1p and 19q [16]. *CIC* resides within the portion of 19q that is lost. The observation that the remaining copy of *CIC* frequently harbours a somatic mutation that either truncates the protein or results in a loss of DNA binding activity supports the notion that CIC may have a tumour suppressor role in ODG. Moreover, the invariable co‐occurrence of *CIC* and *IDH1/2* mutations in ODG is compatible with the notion that a functional relationship exists between these two alterations in conferring a selective advantage to cells in ODG progression. However, both CIC's putative tumour suppressor role and its connection with mutant IDH proteins remain poorly understood.

Recent studies have identified several chromatin modifier proteins to be interactors with CIC, indicating a linkage between the epigenome and CIC's function [2, 11]. Additionally, the *IDH1/IDH2* mutations characteristic of ODG tumours where *CIC* mutations are found result in the overproduction of 2‐hydroxyglutarate (2‐HG), which has the downstream consequence of widespread hypermethylation of CpG sites and histone tail residues [17, 18, 19]. Considering the emerging link between CIC and chromatin modifiers, we posited that *CIC* and *IDH* mutations may functionally collaborate to dysregulate the transcriptome and/or the epigenome. We thus analysed genome‐wide profiles of RNA expression, DNA methylation, and selected histone modifications in *CIC‐*wild type (*CIC‐*WT) and *CIC*‐knockout (*CIC*‐KO) cell lines, in the presence and absence of IDH1‐R132H expression, seeking new insight into how CIC loss and mutant IDH protein expression might interact to promote ODG.

## Materials and methods

### Ethics statement

The work presented here was approved by the UBC BC Cancer Research Ethics Board (H19‐030103, H08‐02838).

### Generation of isogenic *CIC* wild‐type and knockout immortalized astrocyte cell line models using CRISPR‐Cas9

The *E6/E7/hTERT* + *IDH1*‐WT and *E6/E7/hTERT* + *IDH1*‐R132H human astrocyte cell lines were obtained from Applied Biological Materials Inc (Richmond, BC, Canada; T3022 and T3023). With the *E6/E7/hTERT* + *IDH1*‐R132H cell line, we observed gradual loss of IDH1‐R132H protein expression over serial passages. Single clone screens involving iterative western blotting over multiple passages were conducted to obtain a monoclonal cell line that stably expressed IDH1‐R132H. In this work, the *E6/E7/hTERT* + *IDH1*‐WT cell line is referred to as *CIC*‐WT (IDH1‐WT) and the monoclonal *E6/E7/hTERT* + *IDH1*‐R132H cell line is referred to as *CIC*‐WT (IDH1‐R132H).

**Figure 1:**
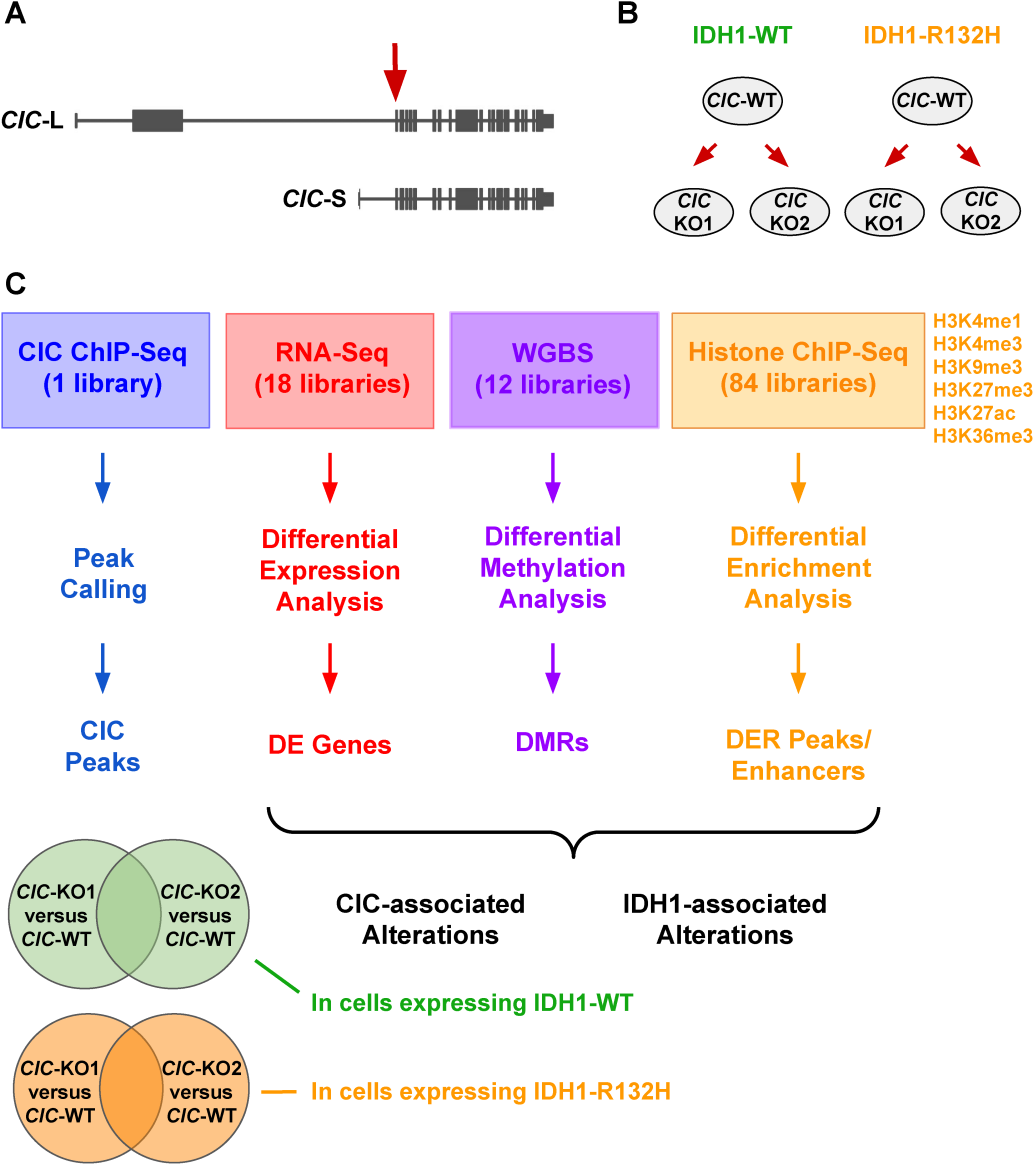
Characterization of the transcriptional and epigenomic consequences of *CIC*‐KO and IDH1‐R132H protein expression. (A) The target site of the CRISPR‐Cas9 sgRNA used to generate *CIC*‐KO cell lines is located at the first commonly shared exon between the two *CIC* isoforms (*CIC*‐L: long isoform; *CIC*‐S: short isoform). (B) The experimental models used in this study: from immortalized human astrocyte cell lines expressing either IDH1‐WT or IDH1‐R132H, two independent *CIC*‐KO cell lines were obtained using CRISPR‐Cas9. (C) The ‐omic datasets and analytical workflow involved in characterizing the transcriptomic and epigenomic consequences of CIC loss and mutant IDH1 expression (also see Materials and methods section). From each differential analysis, CIC‐associated and IDH1‐associated alterations were identified by comparing *CIC*‐KO cells with their *CIC*‐WT parental counterparts, and *CIC*‐WT (IDH1‐R132H) cells with *CIC*‐WT (IDH1‐WT) cells, respectively. Since there were two *CIC*‐KO cell lines in each *IDH1* context, we considered CIC‐associated alterations as those that consistently appeared in both *CIC*‐KO cell lines (i.e. the intersection of the Venn diagrams) within each respective IDH1 background. DE, differentially expressed; DER, differentially enriched; DMR, differentially methylated region.

CRISPR‐Cas9 sgRNA sequences were designed to target exon 2 of *CIC* (chr19:42 791 005–42 791 024) [20] and used to generate two *CIC*‐KO cell lines each from the *CIC*‐WT (IDH1‐WT) and *CIC*‐WT (IDH1‐R132H) parental lines (Figure 1). The absence of CIC and stable IDH1‐R132H protein expression were confirmed using western blotting of whole‐cell lysates (supplementary material, Figure S1). All cell lines were cultured in Dulbecco's Modified Eagle's Medium supplemented with 10% (v/v) heat‐inactivated fetal bovine serum (Life Technologies, Carlsbad, CA, USA) and incubated in a humidified, 37 °C, 5% CO_2_ incubator.

### Whole transcriptome library construction and sequencing

Details are presented in Supplementary materials and methods.

### Differential expression analysis

Raw read counts were mapped onto Ensembl 75 gene annotations using JAGuaR [21]. DESeq2 [22] v.1.8.2 was used to conduct independent differential expression analyses between each *CIC*‐KO line and its *CIC*‐WT counterpart, and between the *CIC*‐WT (IDH1‐R132H) and *CIC*‐WT (IDH1‐WT) lines. Differential expression analysis results are presented in supplementary material, Table S1, including whether each differentially expressed (DE) gene was also identified as DE in *CIC*‐mutant ODGs compared with *CIC*‐WT ODGs [20]. DE genes were considered statistically significant if they met a Benjamini–Hochberg‐adjusted *P* value (*q*‐value) of 0.05 and were considered CIC‐associated if they met this threshold and had concordant directionality.

### Functional enrichment analysis

CIC‐associated DE genes within each IDH1 context were submitted separately for pathway enrichment analysis using Metascape [23]. For IDH1‐associated DE genes (*n* = 6044), those with a fold‐change ≥ 2 were submitted (*n* = 2722), since Metascape restricts gene lists for pathway enrichment analysis to 3000 genes.

### CIC ChIP‐seq analysis

Methods for CIC chromatin immunoprecipitation and sequencing, including processing of CIC ChIP‐seq data and peak calling, are described in detail in Supplementary materials and methods. CIC peaks derived from our dataset were assessed for overlap (≥1 bp) with CIC peaks from a published CIC ChIP‐seq dataset [2]. The 150 most significant peaks in our dataset were deemed high‐confidence CIC peaks based on the inflection point at which the presence of reproducibly identified peaks increased in relation to peak rank by statistical significance (supplementary material, Figure S2).

Genomic features of high‐confidence CIC peaks were obtained using ChIPseeker [24]. *De novo* motif enrichment analysis was conducted on high‐confidence CIC peaks centred on their summits (the coordinate at which fold‐enrichment of ChIP read coverage relative to its matched control was greatest) using HOMER [25] v.4.9.1, with the size parameter set to 200 bp as recommended for identifying primary and co‐enriched motifs for transcription factors (TFs) (http://homer.ucsd.edu/homer/ngs/peakMotifs.html).

### Histone modification ChIP‐seq analysis

Methods for histone modification ChIP, library construction and sequencing, and processing of histone modification ChIP‐seq data, including the identification of peaks and enhancer regions, are described in Supplementary materials and methods.

CIC‐associated and IDH1‐associated differentially enriched (DER) peaks were identified using DESeq2, analogously to the identification of DE genes. DER peaks were required to meet a *q*‐value threshold of 0.05 and a fold‐change ≥ 2 to be considered significant, and additionally required directional concordance between both *CIC*‐KO replicate cell lines for CIC‐associated DER peaks. DER peaks were annotated with their associated genomic feature and nearest gene using ChIPseeker.

Enhancers were labelled as DER if an overlap was present with at least one H3K4me1 or H3K27ac DER peak. The nearest genes associated with DER enhancers were considered to be putative targets of such enhancers. *De novo* motif analysis was performed on downregulated and upregulated DER enhancers using HOMER, with the size parameter set to 500 bp as recommended for histone marked regions (http://homer.ucsd.edu/homer/ngs/peakMotifs.html) and otherwise default parameters.

### Differential methylation analysis

Methods for whole genome bisulphite sequencing and data processing, including differential methylation analysis, are described in Supplementary materials and methods. Differentially methylated regions (DMRs) were identified using Defiant [26] with default parameters. DMRs between replicate *CIC*‐KO cell lines were assessed for both overlap (≥1 bp) and concordant directionality, and were considered to be CIC‐associated if they met these criteria.

## Results

### Transcriptome and epigenome profiles of *CIC*‐KO cell lines expressing IDH1‐WT and IDH1‐R132H

To investigate the effects of CIC loss and IDH1‐R132H expression on transcriptomes and epigenomes, we performed whole‐transcriptome sequencing (RNA‐seq), whole‐genome bisulphite sequencing (WGBS), and chromatin immunoprecipitation followed by DNA sequencing (ChIP‐seq) for six different histone modifications (H3K4me1, H3K4me3, H3K27ac, H3K27me3, H3K9me3, H3K36me3) to analyse *CIC*‐WT (IDH1‐WT), *CIC*‐KO (IDH1‐WT), *CIC*‐WT (IDH1‐R132H), and *CIC*‐KO (IDH1‐R132H) cell lines. In each IDH1 context, two independent *CIC*‐KO lines were derived and are individually referred to as *CIC*‐KO1 and *CIC*‐KO2 throughout this study. For each ‐omic dataset, we identified alterations present in *CIC*‐KO cells relative to *CIC*‐WT cells (i.e. CIC‐associated alterations) in both IDH1‐WT and mutant contexts. In a similar manner, we identified IDH1‐associated differences by comparing *CIC*‐WT (IDH1‐WT) with *CIC*‐WT (IDH1‐R132H). We also conducted CIC ChIP‐seq to identify CIC binding sites in the IDH1‐WT line. The experimental models, datasets, and analytical approaches used in this study are outlined in Figure 1.

### *CIC* knockout and mutant IDH1 expression have overlapping consequences at the level of differentially expressed genes and pathways

**Figure 2:**
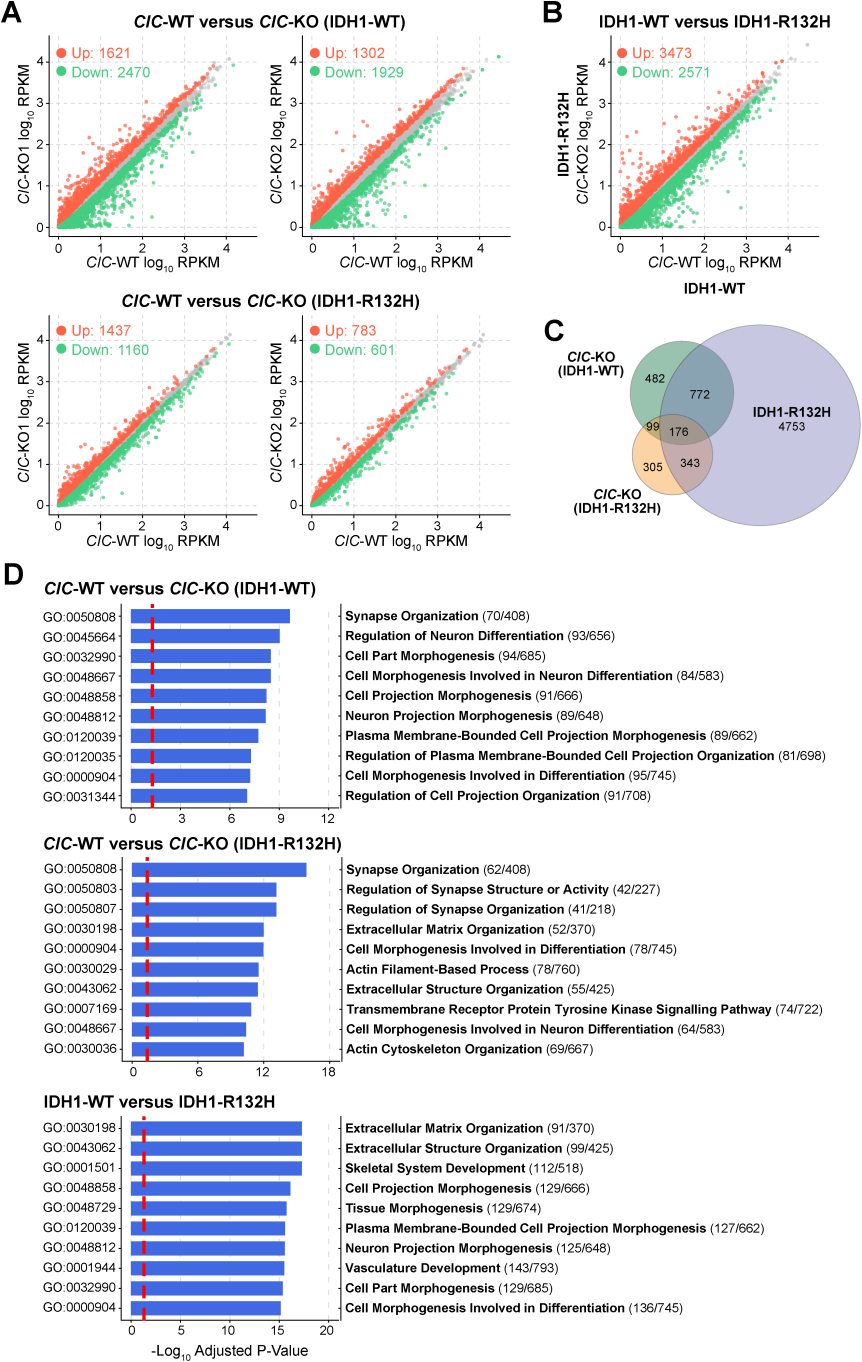
Differentially expressed genes are shared between *CIC*‐KO and IDH1‐R132H‐expressing cells. (A) Scatter plots displaying average gene expression [log_10_ reads per kilobase million (RPKM)] across three replicates for each *CIC*‐KO cell line (*y*‐axis) against its *CIC*‐WT counterpart (*x*‐axis). Each dot represents a protein‐coding gene and is coloured red if the gene was upregulated, green if it was downregulated, and grey if it did not meet the significance threshold (*q* < 0.05). (B) Same plot as in A but comparing gene expression between the *CIC*‐WT (IDH1‐R132H) parental cell line (*y*‐axis) and the *CIC*‐WT (IDH1‐WT) parental cell line (*x*‐axis) to identify IDH1‐associated genes. (C) Venn diagram displaying the intersections of all DE analyses. Only the genes significantly and concordantly DE between replicate *CIC*‐KO cell lines were considered to be CIC‐associated DE genes. For IDH1‐associated DE genes, all of those that were significant (*q* < 0.05) between *CIC*‐WT (IDH1‐WT) and *CIC*‐WT (IDH1‐R132H) were considered. (D) Top ten gene ontology terms enriched within each DE gene set. Dashed lines indicate the threshold for statistically significant enrichment (*q* = 0.05). The numbers beside each term name denote the number of DE genes over the total number of genes within the corresponding gene ontology term.

We conducted differential expression analyses to compare gene expression levels between each *CIC*‐KO cell line and its parental *CIC*‐WT counterpart (see Materials and methods). The number of up‐ and down‐regulated protein‐coding genes obtained from each *CIC*‐KO versus *CIC*‐WT comparison is presented in Figure 2A. Genes that were significantly DE with consistent direction in both *CIC*‐KO cell lines in each IDH1 context (CIC‐associated DE genes) were considered for further analysis. These comprised 1529 genes [661 (43.2%) up and 868 (56.8%) down] in IDH1‐WT cells and 923 genes [501 (54.3%) up and 422 (45.7%) down] in IDH1‐R132H cells. Among those consistently DE in *CIC*‐KO cells, regardless of IDH1 status, were *ETV1*, *ETV4*, *ETV5*, and *GPR3*, which is consistent with their known status as direct targets of CIC repression as demonstrated in several cell/tissue types (e.g. 20, 27, 28, 29). Other genes whose promoters were previously confirmed to be bound by CIC in HEK cells (*ETV1*, *DUSP4*, *GPR3*, *SPRY4*, *SHC3*, and *SHC4* [20]) were also generally upregulated (supplementary material, Figure S3 and Table S1). We also found that 66 and 46 CIC‐associated DE genes identified in IDH1‐WT and IDH1‐R132H cell lines, respectively, overlapped with genes previously found to be DE in *CIC*‐WT versus *CIC*‐deficient primary ODGs [20] (supplementary material, Table S1). This relatively low overlap is consistent with CIC‐associated DE genes being largely context‐specific, as previously demonstrated [30].

Significantly DE genes in *CIC*‐WT (IDH1‐R132H) relative to *CIC*‐WT (IDH1‐WT) (i.e. IDH1‐associated DE genes) comprised a greater number of genes [3473 (57.5%) up and 2571 (42.5%) down] compared with CIC‐associated DE genes (Figure 2B). IDH1‐associated DE genes significantly overlapped with CIC‐associated DE genes in both IDH1 backgrounds (Figure 2C; *p* < 2e‐161 and *p* < 1.5e‐76, respectively; Fisher's exact test), indicating a considerable overlap between the transcriptional consequences of CIC loss and IDH1‐R132H expression.

To glean insights into CIC‐associated transcriptional alterations at the level of biological pathways, we conducted functional enrichment analyses of DE genes (see Materials and methods). Consistent with previous associations made between CIC and central nervous system (CNS) development [9, 10, 11], pathways related to neuron differentiation and synapse formation were among the most significantly enriched terms for CIC‐associated DE genes in both IDH1 backgrounds (Figure 2D and supplementary material, Table S2). These same terms were among the top enriched pathways for IDH1‐associated DE genes, indicating an overlap between the consequences of *CIC*‐KO and IDH1‐R132H expression at the level of biological processes in addition to the overlap at the level of DE genes noted previously.

### CIC ChIP‐seq identifies neurodevelopmental genes as potentially novel direct target genes of CIC

**Figure 3:**
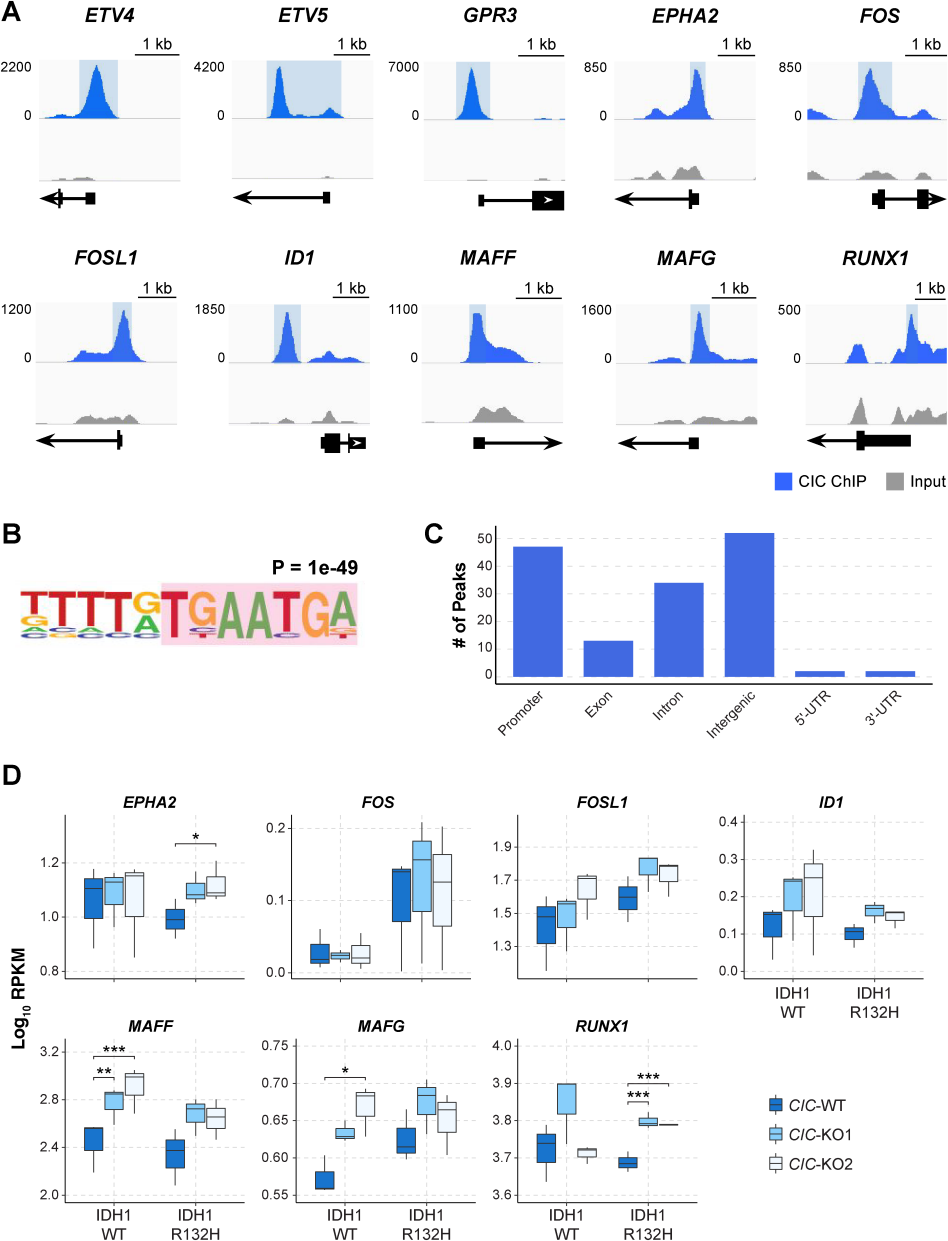
Genome‐wide characterization of CIC occupancy reveals novel candidate binding sites at neurodevelopmental genes. (A) CIC ChIP‐seq (blue) and matched input (grey) RPKM coverage in 10‐bp bins at a subset of high‐confidence peaks in proximity to gene promoters, visualized on the Integrative Genomics Viewer (IGV). The portion of the track highlighted in blue indicates the region called as a CIC peak. Numbers on the left refer to the RPKM scale of both the ChIP and the input coverage tracks for each gene. RefSeq gene models are shown at the bottom, with strand orientation denoted by the direction of the arrowheads. (B) The most significantly enriched *de novo* motif identified within CIC peaks using HOMER software. A portion of the known CIC consensus binding sequence is highlighted in pink. (C) Distribution of high‐confidence CIC peaks in relation to their association with genomic features. (D) Expression levels (RPKM) across all cell lines of selected genes for which a reproducibly identified CIC peak was present near their TSS. *q < 0.05, **q < 0.005, ***q < 0.0005.

To help identify candidate direct CIC targets among the list of CIC‐associated DE genes, we performed CIC ChIP‐seq on the *CIC*‐WT (IDH1‐WT) cell line. We focused on the 150 most significant CIC peaks based on the overlap of these peaks with an independently generated CIC ChIP‐seq peak set [2] (supplementary material, Figure S2 and Table S3; Materials and methods). As expected, among these 150 peaks were those in close proximity to the transcriptional start sites (TSSs) of known CIC target genes, such as *DUSP4*, *ETV4*, *ETV5*, *GPR3*, *SPRY4*, and *PLK3* (Figure 3A and supplementary material, Table S3). Moreover, the most significantly enriched motif (*p* < 1e‐49) within these 150 peaks contained the known CIC consensus binding site [2, 31] (Figure 3B). Thus, we refer to these 150 reproducibly identified peaks as high‐confidence CIC peaks. Consistent with published results [2], over half of the high‐confidence CIC peaks were located in introns and intergenic regions (Figure 3C), indicating that CIC might have regulatory roles at regions other than TSSs.

In addition to known CIC target genes, high‐confidence peaks were found at the promoters of *FOS*, *FOSL1*, *MAFF*, *MAFG*, *EPHA2*, *ID1*, and *RUNX1* (Figure 3A). Of these genes, *EPHA2*, *MAFF*, *MAFG*, and *RUNX1* were significantly (*q* < 0.05) upregulated in at least one *CIC*‐KO cell line compared with its *CIC*‐WT counterpart (Figure 3D). *FOSL1* and *ID1* also generally exhibited increased transcript levels in *CIC*‐KO cells compared with *CIC*‐WT cells, although the differences were not statistically significant (Figure 3D). These observations support the notion that these genes may be previously unexplored direct targets of CIC‐mediated transcriptional repression.

### *CIC*‐KO is associated with dysregulation of enhancers near neurodevelopmental genes

To assess the effects of CIC loss and IDH1‐R132H expression on histone modification patterns, we used ChIP‐seq to profile H3K4me1, H3K4me3, H3K27ac, H3K27me3, H3K9me3, and H3K36me3 in our cell line models. Consistent with previous reports associating mutant IDH and increased histone methylation (e.g. ref 19), the cell lines expressing IDH1‐R132H displayed greater genomic enrichment of H3K4me1, H3K4me3, and H3K27me3 compared with those expressing IDH1‐WT (supplementary material, Figure S4). To identify specific changes in chromatin state [i.e. differentially enriched (DER) peaks] associated with different CIC and IDH1 states, we compared read counts within peaks across mutant and wild‐type states (supplementary material, Figure S5A). Similar to the RNA‐seq analysis (Figure 2C), a considerable proportion of CIC‐associated DER peaks were also IDH1‐associated, while the overlap between CIC‐associated DER peaks in the two IDH1 backgrounds was comparatively small (supplementary material, Figure S5B). This indicates that the majority of changes in the chromatin landscape attributed to CIC loss may be additionally influenced by IDH1‐R132H expression.

**Figure 4:**
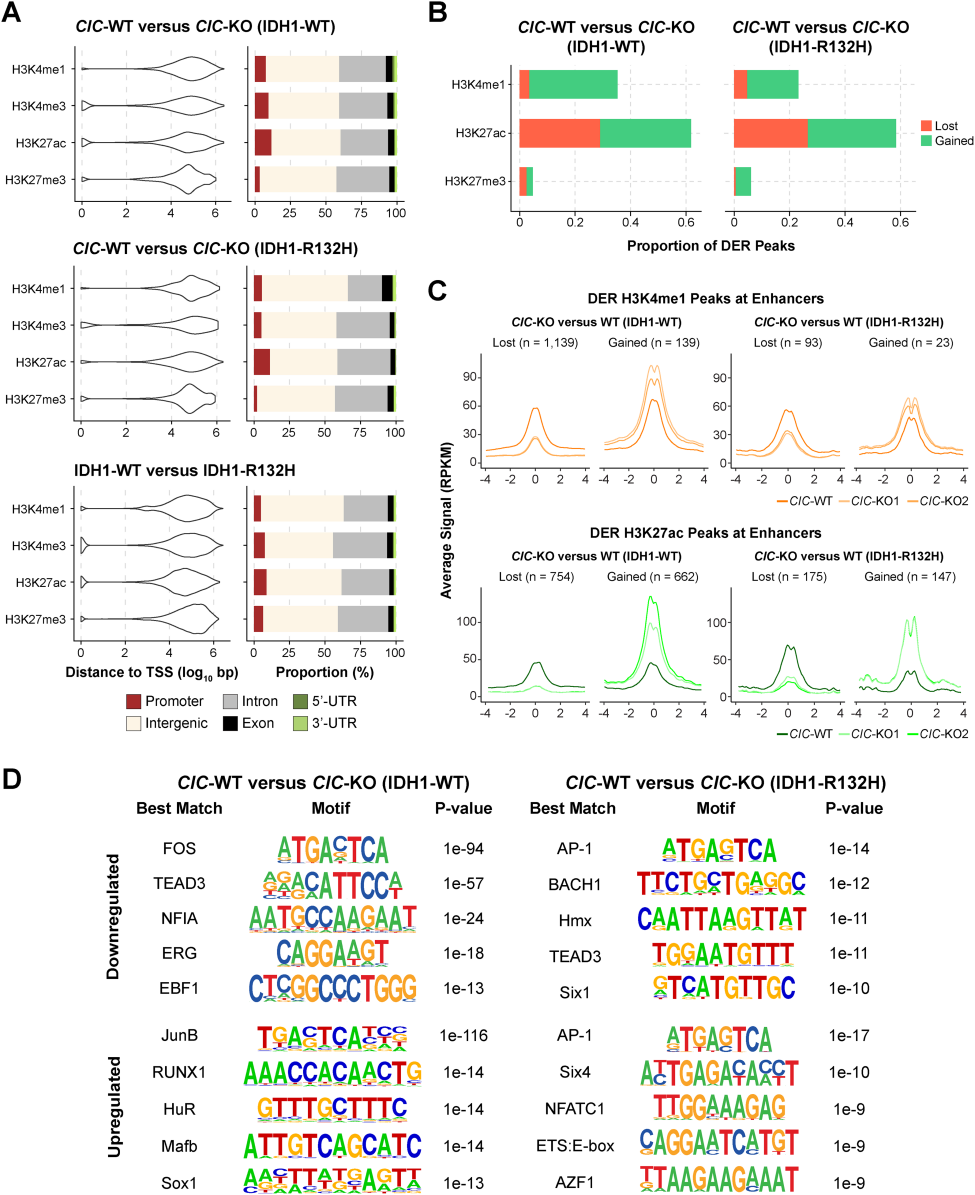
CIC loss is associated with enhancer dysregulation. (A) Distribution of DER peaks for H3K4me1, H3K4me3, H3K27ac, and H3K27me3 with respect to their distance to the nearest TSS (violin plot, left) and their associated genomic feature (bar plot, right). (B) Proportions of CIC‐associated H3K4me1, H3K27ac, and H3K27me3 DER peaks (of all DER peaks for each mark) found at enhancer regions. Colours represent the proportion of DER peaks that exhibit loss in *CIC*‐KO cells (red) or gain in *CIC*‐KO cells (green). (C) Signal profiles for H3K4me1 and H3K27ac at CIC‐associated DER enhancers. Profiles represent RPKM in 100‐bp bins, averaged across all up‐ or down‐regulated DER enhancers for each cell line. (D) Top five significantly enriched *de novo* motifs within CIC‐associated DER enhancers in each IDH1 context. Motifs are presented with their associated *P* values and the transcription factor with the closest matching motif identified using HOMER.

CIC‐associated DER H3K4me1, H3K4me3, H3K27me3, and H3K27ac peaks were predominantly located more than 10 kb away from a TSS and at introns and intergenic regions (Figure 4A), indicating that CIC loss may primarily affect distal regulatory elements. A similar result was observed regarding IDH1‐associated DER peaks (Figure 4A). The observation that most DER peaks were not proximal to TSSs, together with the presence of candidate CIC target genes whose functions are linked to enhancer activity such as *FOS*, *FOSL1*, and *ETV5* [32, 33, 34], led us to investigate chromatin state changes at enhancer regions. Approximately 36% and 23% of CIC‐associated H3K4me1 DER peaks and 62% and 58% of CIC‐associated H3K27ac DER peaks were found at enhancer regions in the IDH1‐WT and IDH1‐R132H contexts, respectively (Figure 4B; Materials and methods). The H3K4me1 and H3K27ac coverage profiles at enhancers overlapping a DER H3K4me1 and/or H3K27ac peak (henceforth referred to as DER enhancers) confirmed the differences in mean coverage between *CIC*‐WT cells and their *CIC*‐KO counterparts, primarily around the centre of enhancer regions (Figure 4C).

Motif enrichment analysis within CIC‐associated DER enhancers revealed that motifs related to the AP‐1 complex, which encompasses the CIC candidate targets *FOS* and *FOSL1*, were the most significantly enriched across all DER enhancers (Figure 4D). Motifs that most closely resembled ETS‐family transcription factors (TFs; ERG and ETS:E‐box), to which *ETV1/4/5* belong, also emerged. Furthermore, motifs matching those of additional candidate CIC targets, namely *RUNX1*, *MAFB*, and *BACH1*, were among the top five most significantly enriched (Figure 4D). Together, these results illustrate that CIC loss may indirectly lead to enhancer disruption through the de‐repression of *ETV* genes and other putative direct CIC targets.

**Figure 5:**
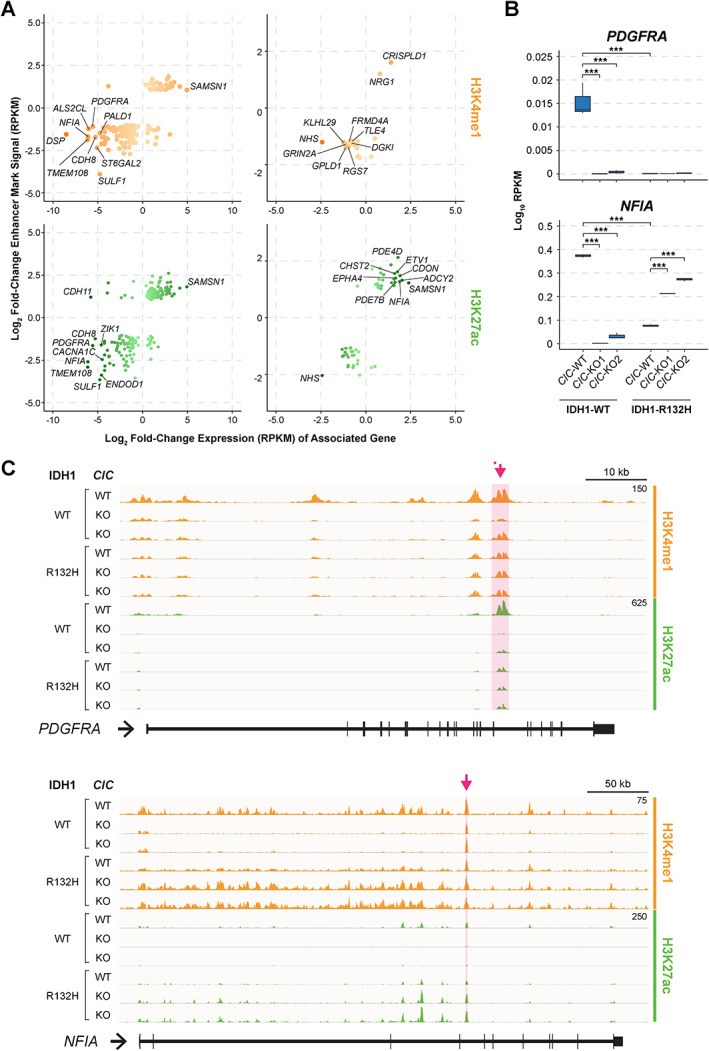
Enhancers at *PDGFRA* and *NFIA* are dysregulated in association with CIC and IDH1 status. (A) Change in ChIP signal (log_2_ fold‐change RPKM, *CIC*‐KO versus WT) for H3K4me1 and H3K27ac peaks at CIC‐associated DER enhancers (*y*‐axis) versus change in gene expression (log_2_ fold‐change RPKM, *CIC*‐KO versus WT) of associated genes (*x*‐axis) in IDH1‐WT and mutant contexts. Colour intensity of points corresponds to absolute fold‐change in gene expression (darker for greater fold‐changes). The top ten genes that displayed the greatest absolute changes in gene expression are labelled in each plot. (B) *PDGFRA* and *NFIA* gene expression (log_10_ RPKM) across all cell lines. ***q < 0.0005. (C) IGV tracks displaying H3K4me1 and H3K27ac signal (RPKM) across all cell lines at the *PDGFRA* (top) and *NFIA* (bottom) gene loci. The RPKM signal range for each mark at each locus was set at the same scale in RPKM across all cell lines (numbers at the top right corner of each set of tracks). Regions highlighted in pink under the red arrows represent the enhancer regions identified to possess a DER H3K4me1 and/or H3K27ac peak.

Consistent with the association of H3K4me1 and H3K27ac with active enhancers, there was a clear positive correlation between both H3K4me1 and H3K27ac enrichment at enhancers and the expression of their closest genes (Figure 5A). Putative gene targets of such enhancers included *CDH8*, *TMEM108*, *PDGFRA*, and *NFIA* in the IDH1‐WT model. In the IDH1‐R132H model, prominent DER enhancer‐associated genes included *NRG1*, *EPHA4*, *ETV1*, and *NFIA*. Expression of *PDGFRA*, a glioblastoma (GBM)‐associated gene [35], was observed to be lower in comparisons of both *CIC*‐KO to *CIC*‐WT cells and IDH1‐R132H to IDH1‐WT cells, indicating that both CIC loss and the expression of IDH1‐R132H independently resulted in reduced expression of *PDGFRA* (Figure 5B). Interestingly, an enhancer within *PDGFRA* displayed a lower enrichment of active marks (H3K4me1 and H3K27ac) in the same cell lines that exhibited lower *PDGFRA* expression, suggesting that the loss in *PDGFRA* expression was due to the inactivation of this enhancer (Figure 5C). An intragenic enhancer within *NFIA*, a regulator of both gliogenesis and gliomagenesis [36, 37], exhibited a reduction of H3K27ac in *CIC*‐KO cells compared with their *CIC*‐WT counterparts and in *CIC*‐WT (IDH1‐R132H) compared with *CIC*‐WT (IDH1‐WT) cells, again illustrating the independent impacts of *CIC*‐KO and IDH1‐R132H expression in reducing the enrichment of active marks at the same enhancer. Intriguingly, however, *CIC*‐KO cells that also expressed IDH1‐R132H displayed a gain of H3K27ac, which we interpret as a potential reactivation of this enhancer (Figure 5C). This intriguing pattern of enhancer dysregulation was also accompanied by concurrent changes in *NFIA* gene expression (Figure 5B). In summary, these examples highlight the impacts of *CIC*‐KO and IDH1‐R132H on enhancers at genes associated with CNS tumours and, regarding *NFIA*, illustrate an interesting case of an enhancer whose activity is differentially regulated by CIC loss in an IDH1‐dependent fashion.

### Analysis of differentially methylated regions identifies CIC and IDH1‐associated changes in the DNA methylation landscape

**Figure 6:**
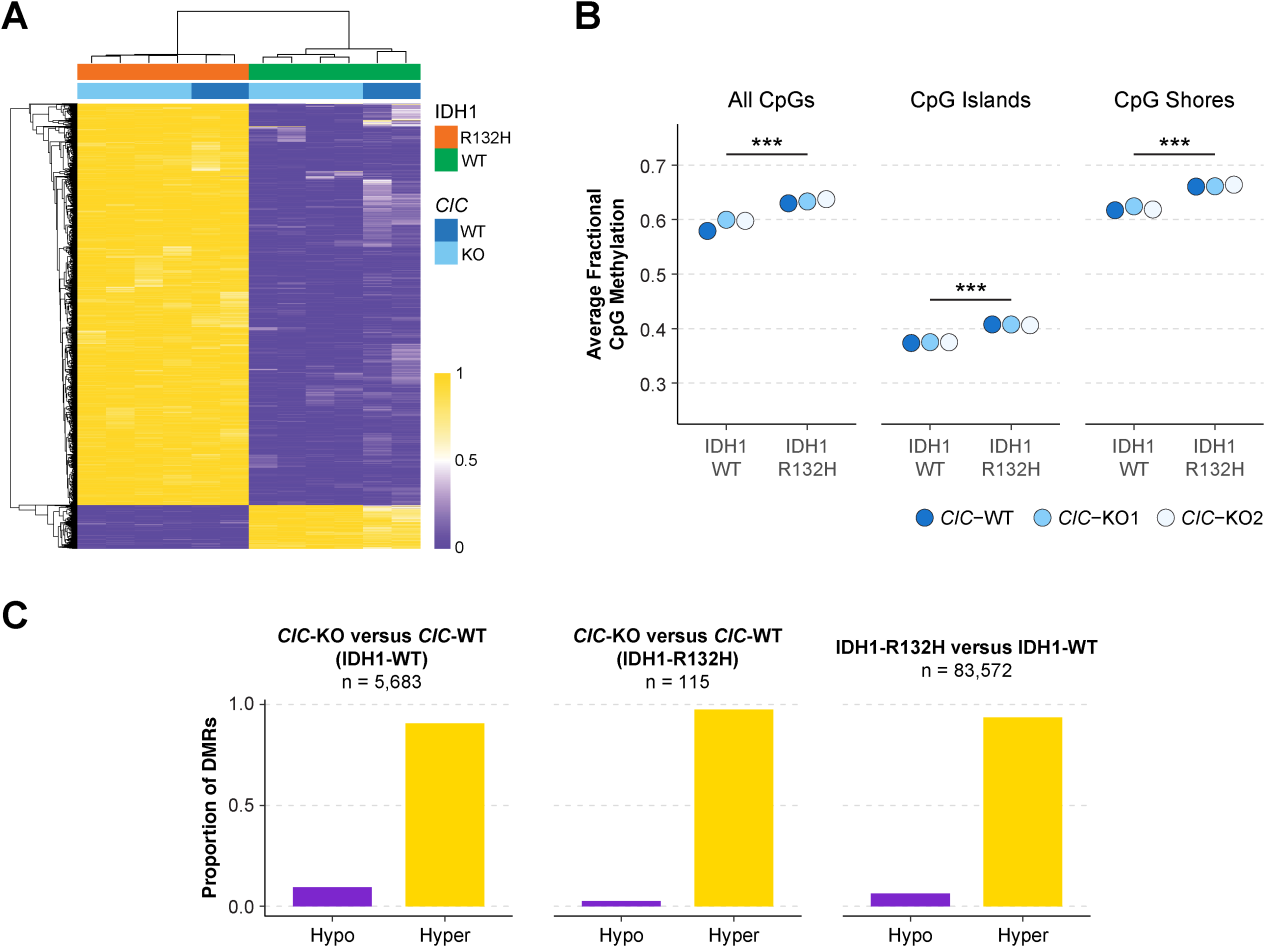
Both CIC loss and IDH1‐R132H are associated with DNA hypermethylation. (A) Heatmap displaying fractional methylation of the top 10 000 most variably methylated CpG sites across all samples. Samples and CpGs were clustered using unsupervised hierarchical clustering. (B) Average fractional methylation for CpGs with greater than five raw read coverage across all CpGs, CpG islands, and CpG shores. ***p < 0.0005 (Mann–Whitney *U*‐test). (C) Proportions of hypomethylated and hypermethylated CIC and IDH1‐associated DMRs.

To investigate the effects of CIC loss or IDH1‐R132H on DNA methylation, we generated WGBS libraries from our cell line models. In agreement with IDH1‐R132H expression being linked to global hypermethylation [19], the majority of the top 10 000 most variably methylated CpG sites exhibited hypermethylation in cells expressing IDH1‐R132H (Figure 6A). Moreover, mean genome‐wide CpG methylation, as well as methylation within CpG islands and CpG shores, was significantly higher (*p* < 0.0005) in all cell lines expressing IDH1‐R132H compared with those expressing IDH1‐WT (Figure 6B). To identify regions of DNA methylation that were affected by CIC loss or IDH1‐R132H, we conducted a differentially methylated region (DMR) analysis using Defiant [26] (Materials and methods). Of the 43 302 DMRs (*CIC*‐KO versus WT) identified in *CIC*‐KO1 (IDH1‐WT) and the 42 259 DMRs identified in *CIC*‐KO2 (IDH1‐WT), only 5683 (~13%) were in common and had consistent directionality. A much lower number of DMRs were identified between *CIC*‐KO and WT cells expressing IDH1‐R132H: 2605 in *CIC*‐KO1 (IDH1‐R132H), 1835 in *CIC*‐KO2 (IDH1‐R132H), and only 115 (<6.3%) in common and concordant between both. Unsurprisingly, many more IDH1‐associated DMRs were identified, totalling 83 572.

Strikingly, CIC‐associated DMRs almost exclusively involved increased DNA methylation, as was expected and observed with IDH1‐associated DMRs (Figure 6C). Considering CIC's established role as a transcriptional repressor, the prominence of hypermethylated DMRs relative to hypomethylated DMRs in *CIC*‐KO cells was unexpected. However, we found no association between CIC binding sites and these DMRs (supplementary material, Figure S6), and also found no correlation between DMRs and the expression of their closest genes (supplementary material, Figure S7A,B). These observations illustrate that CIC‐associated DMRs likely arose independently of CIC binding and appeared to have minimal impact on gene expression.

## Discussion

Recent studies have indicated a regulatory role for CIC in neurodevelopment, in which CIC loss resulted in neural maturation defects [11], the promotion of EGF‐independent NSC proliferation [10], and the expansion of NSCs and oligodendrocyte precursor cells (OPCs) [9]. These studies support the notion that functional CIC may be important in the maintenance of NSC quiescence, and that CIC loss can promote increased proliferation and partial commitment towards the oligodendrocyte lineage. Moreover, recurrent *CIC* mutations are found in gliomas (exclusively in those possessing an *IDH1/2* mutation), implicating CIC as a potential tumour suppressor in this cancer type. In this study, we characterized the effects of *CIC*‐KO on global gene expression, histone modification profiles, and DNA methylation patterns in IDH1‐WT and IDH1‐R132H backgrounds, including analyses of reproducible CIC binding sites.

In IDH1‐WT cells, we identified novel or previously unexplored candidate CIC target genes, including *RUNX1*, *ID1*, and *EPHA2*. The finding that CIC may directly regulate *RUNX1*, a gene linked to leukaemogenesis [38], may provide mechanistic insight into the reported associations between CIC loss and altered T‐cell development and T‐cell acute lymphoblastic leukaemia (T‐ALL) onset in mice [5, 6, 29]. *RUNX1* also appears to have a pro‐neurogenic role, since its expression was found to correlate with the survival and proliferation of adult neural precursor cells [39]. ID1 has demonstrated roles in GBM tumour progression [40] and the control of NSC quiescence during regenerative neurogenesis [41]. *EPHA2* overexpression was observed to promote glioma stem cell (GSC) invasiveness *in vivo* and promote neurosphere formation *in vitro* [42]. Notably, while *ID1* was upregulated in all *CIC*‐KO lines, *RUNX1* and *EPHA2* were upregulated specifically in *CIC*‐KO (IDH1‐R132H) cells (Figure 3D), suggesting some interplay between CIC loss and mutant IDH1 to promote the activation of *RUNX1* and *EPHA2*. Thus, our identification of *RUNX1*, *ID1*, and *EPHA2* as candidate CIC targets, and their increased expression in *CIC*‐KO cell lines, reveals potential mechanistic insights underpinning the link between CIC and the modulation of neural stem cell fate and ODG. Notably, our CIC ChIP‐seq experiment was only performed in *CIC*‐WT (IDH1‐WT) cells. It is possible that CIC binding may be influenced by the epigenomic consequences of neomorphic IDH1/2. Nevertheless, the increased expression of *RUNX1* and *EPHA2* observed in *CIC*‐KO (IDH1‐R132H) indicates that these genes are potentially direct or indirect targets of CIC in an IDH‐mutant context.

Based on our data, the consequences of CIC loss on histone modifications appeared to largely impact enhancers whose differential histone modification profiles may be ascribed to the differential expression of direct CIC target genes. Supporting this notion, we observed a significant enrichment of motifs matching those of known and candidate CIC targets such as *ETV1/4/5*, *RUNX1*, *MAFF/G*, and *FOS/FOSL1* at DER enhancers (Figure 4D). DER enhancer‐associated genes, including *PDGFRA* [34] and *NFIA* [35, 36], have been linked to the tumourigenicity of glioma models and/or neural progenitor cell fate decisions. Ablation of *PDGFRA* was shown to lead to precocious differentiation of OPCs in the developing spinal cord [43]. The relationship between CIC and *PDGFRA* dysregulation may therefore be of relevance in the context of CIC's function in neural cell fate specification and warrants further investigation. Interestingly, our results were in contrast to published observations of increased *PDGFRA* expression in *IDH*‐mutant gliomas and glioma cell lines [44]. The pattern of dysregulation of a genic enhancer in *NFIA* and its expression showcased a striking phenomenon in which the expression of IDH1‐R132H appeared to have reversed the effect of CIC loss. Furthermore, *NFIA* was found to be upregulated in *CIC*‐mutant cells compared with *CIC*‐wild type cells in a single‐cell gene expression analysis of a primary ODG [45], indicating that the increased *NFIA* expression that we observed in *CIC*‐KO (IDH1‐R132H) cells may be relevant in a primary tumour context. Together with NFIA's demonstrated role in gliomagenesis, our finding supports the view that *NFIA* dysregulation may partially underlie the synergistic relationship between CIC loss and mutant IDH1 in ODG pathology.

The NHA cell line model used in this study presents some caveats. Firstly, it exhibits impaired p53 and RB function as a product of its immortalization [46]. Notably, *TP53* mutations are not found in ODGs but rather in *IDH*‐mutant astrocytomas and are mutually exclusive with *CIC* mutations. It would thus also be of importance to study the consequences of CIC loss in a p53‐proficient background, in regard to CIC's role both in neurodevelopment and in ODG biology. Secondly, the IDH1‐R132H cells overexpress an IDH1‐R132H construct, which has been shown to promote some distinct metabolic features in short‐term culture compared with cells that endogenously express mutant IDH1 [47]. The fact that most of our comparisons were made within IDH1‐WT or IDH1‐R132H cells (comparing *CIC*‐KO with *CIC‐*WT), along with comparisons to CIC‐associated changes in primary ODGs, nevertheless helps to distinguish possible effects of artefacts associated with IDH1‐R132H overexpression. Thirdly, the NHA model is astrocytic in origin; however, while ODGs have traditionally been hypothesized to arise from an oligodendrocytic origin due to their histology, recent single‐cell studies have shown that ODGs and astrocytomas share a common cellular hierarchy [45]. Therefore, our results are relevant to CIC's role in ODG as well as to its role in neurodevelopment.

Neomorphic *IDH1/2* mutations and 1p/19q co‐deletions are the defining features of ODG, while *CIC* alterations are found in ~50–80% of these primary tumours [12, 13]. These mutational frequencies imply an order of events in which *CIC* mutations occur after the *IDH1/2* mutation and 1p/19q co‐deletion. The neomorphic *IDH1* mutation and consequent DNA hypermethylation have been demonstrated to affect the neural developmental hierarchy, specifically in blocking differentiation [48, 49]. Integrating our results and the emerging evidence for CIC being an important mediator of neural/glial cell fate specification, we conceptualize a model in which CIC loss is presumed to lead to the genesis of OPC‐like cells and their expansion is further enabled due to the de‐differentiating influence of neomorphic mutant IDH1 expression. This amplification of self‐renewing cells could provide more opportunities for cancer‐promoting mutations to arise. Overall, our work provides a rationale for future research to examine the functional relationship between CIC loss and neomorphic mutant IDH1 in the context of early neural/glial cell fate.

## Author contributions statement

MAM conceptualized and directed the study. JS generated the CRISPR‐mediated *CIC‐*KO cell lines and participated in cell culturing. SDL prepared all the cell line samples submitted for RNA‐seq, histone ChIP‐seq, and WGBS. VL performed the CIC ChIP experiments and prepared CIC ChIP'ed DNA samples for sequencing. SDL performed all bioinformatics analyses with technical feedback from VL and MAM. SDL wrote the manuscript with feedback from VL and MAM. All the authors read and approved the final manuscript.

## Supporting information


Supplementary materials and methods

**Figure S1.** Confirmation of CIC and IDH1 status in cell line models
**Figure S2.** Number of reproducibly identified CIC peaks versus MACS2 *q*‐value significance
**Figure S3.** Known CIC target genes are overexpressed in *CIC*‐KO cells
**Figure S4.** Comparison of peaks across all cell lines for each histone modification
**Figure S5.** Summary of DER peaks
**Figure S6.** CIC binding is not associated with differential methylation
**Figure S7.** CIC‐associated differential methylation is not associated with differential gene expression

**Table S1.** DESeq2 differential expression analysis results for CIC‐ and IDH1‐associated DE genes

**Table S2.** Metascape pathway enrichment analysis results for CIC‐ and IDH1‐associated DE genes

**Table S3.** ChIPseeker annotations of high‐confidence CIC peaks

## Data Availability

The data that support the findings of this study are being made openly available in the Gene Expression Omnibus under the project title Transcriptomic and Epigenomic Profiles of CIC‐knockout and IDH1‐mutant cells at https://www.ncbi.nlm.nih.gov/geo/query/acc.cgi?acc=GSE189861 (GEO accession GSE189861). Codes used to generate the analyses presented in the paper are publicly available in Github (https://github.com/sdlee94/multi-omic-analysis-of-CIC-KO-and-IDH1-R132H-cells).

## References

[1] Dissanayake K , Toth R , Blakey J , *et al*. ERK/p90^RSK^/14‐3‐3 signalling has an impact on expression of PEA3 Ets transcription factors via the transcriptional repressor capicúa. Biochem J 2011; 433: 515–525. 21087211 10.1042/BJ20101562 PMC3025492

[2] Weissmann S , Cloos PA , Sidoli S , *et al*. The tumor suppressor CIC directly regulates MAPK pathway genes via histone deacetylation. Cancer Res 2018; 78: 4114–4125. 29844126 10.1158/0008-5472.CAN-18-0342 PMC6076439

[3] Lee Y , Fryer JD , Kang H , *et al*. ATXN1 protein family and CIC regulate extracellular matrix remodeling and lung alveolarization. Dev Cell 2011; 21: 746–757. 22014525 10.1016/j.devcel.2011.08.017 PMC3253850

[4] Kim E , Park S , Choi N , *et al*. Deficiency of Capicua disrupts bile acid homeostasis. Sci Rep 2015; 5: 8272. 25653040 10.1038/srep08272 PMC4317698

[5] Park S , Lee S , Lee CG , *et al*. Capicua deficiency induces autoimmunity and promotes follicular helper T cell differentiation via derepression of ETV5. Nat Commun 2017; 8: 16037. 28855737 10.1038/ncomms16037 PMC5510180

[6] Tan Q , Brunetti L , Rousseaux MWC , *et al*. Loss of Capicua alters early T cell development and predisposes mice to T cell lymphoblastic leukemia/lymphoma. Proc Natl Acad Sci U S A 2018; 115: E1511–E1519. 29382756 10.1073/pnas.1716452115 PMC5816173

[7] Lu HC , Tan Q , Rousseaux MW , *et al*. Disruption of the ATXN1–CIC complex causes a spectrum of neurobehavioral phenotypes in mice and humans. Nat Genet 2017; 49: 527–536. 28288114 10.1038/ng.3808 PMC5374026

[8] Lam YC , Bowman AB , Jafar‐Nejad P , *et al*. ATAXIN‐1 interacts with the repressor Capicua in its native complex to cause SCA1 neuropathology. Cell 2006; 127: 1335–1347. 17190598 10.1016/j.cell.2006.11.038

[9] Ahmad ST , Rogers AD , Chen MJ , *et al*. Capicua regulates neural stem cell proliferation and lineage specification through control of Ets factors. Nat Commun 2019; 10: 2000. 31043608 10.1038/s41467-019-09949-6 PMC6494820

[10] Yang R , Chen LH , Hansen LJ , *et al*. Cic loss promotes gliomagenesis via aberrant neural stem cell proliferation and differentiation. Cancer Res 2017; 77: 6097–6108. 28939681 10.1158/0008-5472.CAN-17-1018 PMC5690824

[11] Hwang I , Pan H , Yao J , *et al*. CIC is a critical regulator of neuronal differentiation. JCI Insight 2020; 5: e135826. 10.1172/jci.insight.135826 PMC7253013 32229723

[12] Bettegowda C , Agrawal N , Jiao Y , *et al*. Mutations in *CIC* and *FUBP1* contribute to human oligodendroglioma. Science 2011; 333: 1453–1455. 21817013 10.1126/science.1210557 PMC3170506

[13] Yip S , Butterfield YS , Morozova O , *et al*. Concurrent *CIC* mutations, *IDH* mutations, and 1p/19q loss distinguish oligodendrogliomas from other cancers. J Pathol 2012; 226: 7–16. 22072542 10.1002/path.2995 PMC3246739

[14] The Cancer Genome Atlas Research Network , Brat DJ , Verhaak RG , *et al*. Comprehensive, integrative genomic analysis of diffuse lower‐grade gliomas. N Engl J Med 2015; 372: 2481–2498. 26061751 10.1056/NEJMoa1402121 PMC4530011

[15] Suzuki H , Aoki K , Chiba K , *et al*. Mutational landscape and clonal architecture in grade II and III gliomas. Nat Genet 2015; 47: 458–468. 25848751 10.1038/ng.3273

[16] Louis DN , Perry A , Reifenberger G , *et al*. The 2016 World Health Organization classification of tumors of the central nervous system: a summary. Acta Neuropathol 2016; 131: 803–820. 27157931 10.1007/s00401-016-1545-1

[17] Xu W , Yang H , Liu Y , *et al*. Oncometabolite 2‐hydroxyglutarate is a competitive inhibitor of α‐ketoglutarate‐dependent dioxygenases. Cancer Cell 2011; 19: 17–30. 21251613 10.1016/j.ccr.2010.12.014 PMC3229304

[18] Noushmehr H , Weisenberger DJ , Diefes K , *et al*. Identification of a CpG island methylator phenotype that defines a distinct subgroup of glioma. Cancer Cell 2010; 17: 510–522. 20399149 10.1016/j.ccr.2010.03.017 PMC2872684

[19] Turcan S , Rohle D , Goenka A , *et al*. IDH1 mutation is sufficient to establish the glioma hypermethylator phenotype. Nature 2012; 483: 479–483. 22343889 10.1038/nature10866 PMC3351699

[20] LeBlanc VG , Firme M , Song J , *et al*. Comparative transcriptome analysis of isogenic cell line models and primary cancers links capicua (CIC) loss to activation of the MAPK signalling cascade. J Pathol 2017; 242: 206–220. 28295365 10.1002/path.4894 PMC5485162

[21] Butterfield YS , Kreitzman M , Thiessen N , *et al*. JAGuaR: junction alignments to genome for RNA‐seq reads. PLoS One 2014; 9: e102398. 25062255 10.1371/journal.pone.0102398 PMC4111418

[22] Love MI , Huber W , Anders S . Moderated estimation of fold change and dispersion for RNA‐seq data with DESeq2. Genome Biol 2014; 15: 550. 25516281 10.1186/s13059-014-0550-8 PMC4302049

[23] Zhou Y , Zhou B , Pache L , *et al*. Metascape provides a biologist‐oriented resource for the analysis of systems‐level datasets. Nat Commun 2019; 10: 1523. 30944313 10.1038/s41467-019-09234-6 PMC6447622

[24] Yu G , Wang LG , He QY . ChIPseeker: an R/Bioconductor package for ChIP peak annotation, comparison and visualization. Bioinformatics 2015; 31: 2382–2383. 25765347 10.1093/bioinformatics/btv145

[25] Heinz S , Benner C , Spann N , *et al*. Simple combinations of lineage‐determining transcription factors prime *cis*‐regulatory elements required for macrophage and B cell identities. Mol Cell 2010; 38: 576–589. 20513432 10.1016/j.molcel.2010.05.004 PMC2898526

[26] Condon DE , Tran PV , Lien YC , *et al*. Defiant: (DMRs: easy, fast, identification and ANnoTation) identifies differentially Methylated regions from iron‐deficient rat hippocampus. BMC Bioinformatics 2018; 19: 31. 29402210 10.1186/s12859-018-2037-1 PMC5800085

[27] Okimoto RA , Breitenbuecher F , Olivas VR , *et al*. Inactivation of Capicua drives cancer metastasis. Nat Genet 2017; 49: 87–96. 27869830 10.1038/ng.3728 PMC5195898

[28] Choi N , Park J , Lee JS , *et al*. miR‐93/miR‐106b/miR‐375‐CIC‐CRABP1: a novel regulatory axis in prostate cancer progression. Oncotarget 2015; 6: 23533–23547. 26124181 10.18632/oncotarget.4372 PMC4695135

[29] Simón‐Carrasco L , Graña O , Salmón M , *et al*. Inactivation of Capicua in adult mice causes T‐cell lymphoblastic lymphoma. Genes Dev 2017; 31: 1456–1468. 28827401 10.1101/gad.300244.117 PMC5588927

[30] Wong D , Lounsbury K , Lum A , *et al*. Transcriptomic analysis of CIC and ATXN1L reveal a functional relationship exploited by cancer. Oncogene 2019; 38: 273–290. 30093628 10.1038/s41388-018-0427-5

[31] Jiménez G , Shvartsman SY , Paroush Z . The Capicua repressor – a general sensor of RTK signaling in development and disease. J Cell Sci 2012; 125: 1383–1391. 22526417 10.1242/jcs.092965 PMC3336375

[32] Vierbuchen T , Ling E , Cowley CJ , *et al*. AP‐1 transcription factors and the BAF complex mediate signal‐dependent enhancer selection. Mol Cell 2017; 68: 1067–1082.e12. 29272704 10.1016/j.molcel.2017.11.026 PMC5744881

[33] Pham D , Sehra S , Sun X , *et al*. The transcription factor Etv5 controls T_H_17 cell development and allergic airway inflammation. J Allergy Clin Immunol 2014; 134: 204–214. 24486067 10.1016/j.jaci.2013.12.021 PMC4209254

[34] Kalkan T , Bornelöv S , Mulas C , *et al*. Complementary activity of ETV5, RBPJ, and TCF3 drives formative transition from naive pluripotency. Cell Stem Cell 2019; 24: 785–801.e7. 31031137 10.1016/j.stem.2019.03.017 PMC6509416

[35] Ozawa T , Brennan CW , Wang L , *et al*. *PDGFRA* gene rearrangements are frequent genetic events in *PDGFRA*‐amplified glioblastomas. Genes Dev 2010; 24: 2205–2218. 20889717 10.1101/gad.1972310 PMC2947772

[36] Kang P , Lee HK , Glasgow SM , *et al*. Sox9 and NFIA coordinate a transcriptional regulatory cascade during the initiation of gliogenesis. Neuron 2012; 74: 79–94. 22500632 10.1016/j.neuron.2012.01.024 PMC3543821

[37] Glasgow SM , Carlson JC , Zhu W , *et al*. Glia‐specific enhancers and chromatin structure regulate NFIA expression and glioma tumorigenesis. Nat Neurosci 2017; 20: 1520–1528. 28892058 10.1038/nn.4638 PMC5919190

[38] Sood R , Kamikubo Y , Liu P . Role of RUNX1 in hematological malignancies. Blood 2017; 129: 2070–2082. 28179279 10.1182/blood-2016-10-687830 PMC5391618

[39] Fukui H , Rünker A , Fabel K , *et al*. Transcription factor Runx1 is pro‐neurogenic in adult hippocampal precursor cells. PLoS One 2018; 13: e0190789. 29324888 10.1371/journal.pone.0190789 PMC5764282

[40] Sachdeva R , Wu M , Smiljanic S , *et al*. ID1 is critical for tumorigenesis and regulates chemoresistance in glioblastoma. Cancer Res 2019; 79: 4057–4071. 31292163 10.1158/0008-5472.CAN-18-1357

[41] Rodriguez Viales R , Diotel N , Ferg M , *et al*. The helix‐loop‐helix protein Id1 controls stem cell proliferation during regenerative neurogenesis in the adult zebrafish telencephalon. Stem Cells 2015; 33: 892–903. 25376791 10.1002/stem.1883

[42] Miao H , Gale NW , Guo H , *et al*. EphA2 promotes infiltrative invasion of glioma stem cells *in vivo* through cross‐talk with Akt and regulates stem cell properties. Oncogene 2015; 34: 558–567. 24488013 10.1038/onc.2013.590 PMC4119862

[43] Zhu Q , Zhao X , Zheng K , *et al*. Genetic evidence that *Nkx2*.2 and *Pdgfra* are major determinants of the timing of oligodendrocyte differentiation in the developing CNS. Development 2014; 141: 548–555. 24449836 10.1242/dev.095323 PMC3899813

[44] Flavahan WA , Drier Y , Liau BB , *et al*. Insulator dysfunction and oncogene activation in *IDH* mutant gliomas. Nature 2016; 529: 110–114. 26700815 10.1038/nature16490 PMC4831574

[45] Tirosh I , Venteicher AS , Hebert C , *et al*. Single‐cell RNA‐seq supports a developmental hierarchy in human oligodendroglioma. Nature 2016; 539: 309–313. 27806376 10.1038/nature20123 PMC5465819

[46] Sonoda Y , Ozawa T , Hirose Y , *et al*. Formation of intracranial tumors by genetically modified human astrocytes defines four pathways critical in the development of human anaplastic astrocytoma. Cancer Res 2001; 61: 4956–4960. 11431323

[47] Garrett M , Sperry J , Braas D , *et al*. Metabolic characterization of isocitrate dehydrogenase (IDH) mutant and IDH wildtype gliomaspheres uncovers cell type‐specific vulnerabilities. Cancer Metab 2018; 6: 4. 29692895 10.1186/s40170-018-0177-4 PMC5905129

[48] Turcan S , Fabius AW , Borodovsky A , *et al*. Efficient induction of differentiation and growth inhibition in IDH1 mutant glioma cells by the DNMT inhibitor decitabine. Oncotarget 2013; 4: 1729–1736. 24077826 10.18632/oncotarget.1412 PMC3858559

[49] Rosiak K , Smolarz M , Stec WJ , *et al*. IDH1^R132H^ in neural stem cells: differentiation impaired by increased apoptosis. PLoS One 2016; 11: e0154726. 27145078 10.1371/journal.pone.0154726 PMC4856348

[50] Ramírez F , Dündar F , Diehl S , *et al*. deepTools: a flexible platform for exploring deep‐sequencing data. Nucleic Acids Res 2014; 42: W187–W191. 24799436 10.1093/nar/gku365 PMC4086134

[51] Quinlan AR , Hall IM . BEDTools: a flexible suite of utilities for comparing genomic features. Bioinformatics 2010; 26: 841–842. 20110278 10.1093/bioinformatics/btq033 PMC2832824

[52] Feng J , Liu T , Qin B , *et al*. Identifying ChIP‐seq enrichment using MACS. Nat Protoc 2012; 7: 1728–1740. 22936215 10.1038/nprot.2012.101 PMC3868217

[53] Amemiya HM , Kundaje A , Boyle AP . The ENCODE blacklist: identification of problematic regions of the genome. Sci Rep 2019; 9: 9354. 31249361 10.1038/s41598-019-45839-z PMC6597582

[54] Pellacani D , Bilenky M , Kannan N , *et al*. Analysis of normal human mammary epigenomes reveals cell‐specific active enhancer states and associated transcription factor networks. Cell Rep 2016; 17: 2060–2074. 27851968 10.1016/j.celrep.2016.10.058

