# Supplementary material for: Integrative multi‐omic analysis reveals neurodevelopmental gene dysregulation in CIC ‐knockout and IDH1 ‐mutant cells
Source: J Pathol. 2021 Dec 22;256(3):297–309. doi: 10.1002/path.5835 (PMC9305137; doi:10.1002/path.5835)
Supplement: Supplementary file 1 — Supplementary materials and methods [file PATH-256-297-s002.docx]

**Integrative multi-omic analysis reveals neurodevelopmental gene dysregulation in *CIC*-knockout and *IDH1*-mutant cells**

## SD Lee *et al. J Pathol* DOI: 10.1002/path.5835

## **Supplementary materials and methods**

Reference numbers refer to the main text list

## **Whole transcriptome library construction and sequencing**

To remove cytoplasmic and mitochondrial ribosomal RNA (rRNA) species from total RNA, the NEBNext rRNA Depletion Kit (Human/Mouse/Rat) was used (NEB, Whitby, ON, Canada; E6310X). Enzymatic reactions were set-up in a 96-well plate (Thermo Fisher Scientific, Waltham, MA, USA) on a Microlab NIMBUS liquid handler (Hamilton Robotics, Reno, NV, USA). 100 ng of DNase I‐treated total RNA was hybridized to rRNA probes in a 7.5 μl reaction. Heat-sealed plates were incubated at 95 °C for 2 min, followed by incremental reduction in temperature by 0.1 °C per s to 22 °C (730 cycles). The rRNA in DNA hybrids was digested using RNase H in a 10 µl reaction incubated in a thermocycler at 37 °C for 30 min. To remove excess rRNA probes (DNA) and residual genomic DNA contamination, DNase I was added in a total reaction volume of 25 µl and incubated at 37 °C for 30 min. RNA was purified using RNA MagClean DX beads (Aline Biosciences, Woburn, MA, USA) with 15 min of binding time, 7 min clearing on a magnet followed by two 70% ethanol washes, 5 min to air dry the RNA pellet, and elution in 36 µl of DEPC water. The plate containing RNA was stored at −80 °C prior to cDNA synthesis.

First-strand cDNA was synthesized from the purified RNA (minus rRNA) using the Maxima H Minus First Strand cDNA Synthesis Kit (Thermo Fisher Scientific) and random hexamer primers at a concentration of 8 ng/µl along with a final concentration of 0.04 µg/µl actinomycin D, followed by PCR Clean DX bead purification on a Microlab NIMBUS robot (Hamilton Robotics). The second strand cDNA was synthesized following the NEBNext Ultra Directional Second Strand cDNA Synthesis protocol (NEB) that incorporates dUTP in the dNTP mix, allowing the second strand to be digested using USERTM enzyme (NEB) in the post-adapter ligation reaction and thus achieving strand specificity.

cDNA was fragmented using Covaris LE220 sonication for 100 s (2 × 50 s) at a ‘Duty cycle’ of 30%, 450 Peak Incident Power (W), and 200 Cycles per Burst in a microTUBE Strip (P/N: 520053) to achieve 200–250 bp average fragment lengths. The paired-end sequencing library was prepared following Canada’s Michael Smith Genome Sciences Centre’s strand-specific, plate-based library construction protocol on a Microlab NIMBUS robot (Hamilton Robotics). In brief, the sheared cDNA was subjected to end-repair and phosphorylation in a single reaction using an enzyme premix (NEB) containing T4 DNA polymerase, Klenow DNA polymerase, and T4 polynucleotide kinase, and incubated at 20 °C for 30 min. Repaired cDNA was purified in 96-well format using PCR Clean DX beads (Aline Biosciences), and 3'-A-tailed (adenylated) using Klenow fragment (3' to 5' exo minus) and incubation at 37 °C for 30 min prior to enzyme heat inactivation. PE adapters (Illumina, San Diego, CA, USA) were ligated at 20 °C for 15 min. The adapter-ligated products were purified using PCR Clean DX beads and then digested with USERTM enzyme (1 U/µl; NEB) at 37 °C for 15 min, followed immediately by 13 cycles of indexed PCR using Phusion DNA Polymerase (Thermo Fisher Scientific) and Illumina’s PE primer set. PCR parameters were 98 °C for 1 min followed by 13 cycles of 15 s at 98 °C, 30 s at 65 °C, and 30 s at 65 °C, and then 72 °C for 5 min. The PCR products were twice purified and size-selected using a 1:1 PCR Clean DX beads-to-16 sample ratio, and the eluted DNA quality was assessed using an Agilent DNA1000 Assay (Agilent, Santa Clara, CA, USA) and quantified using a Quant-iT dsDNA High Sensitivity Assay Kit on a Qubit fluorometer (Invitrogen, Waltham, MA, USA). Libraries (3 replicates per cell line) were then pooled and size-corrected using a final molar concentration calculation for Illumina HiSeq2500 sequencing with paired-end 75 base reads.

## **CIC chromatin immunoprecipitation and sequencing**

For each *CIC*-WT (IDH1-WT) cell line replicate, two ~70–80% confluent 15-cm plates were treated with 1% formaldehyde (Sigma, St Louis, MO, USA) in PBS for 12 min with gentle rocking, followed by treatment with 0.125 m glycine (Sigma) for 5 min. Crosslinked cells were combined and pelleted using centrifugation at 1200 rpm for 5 min at room temperature, resuspended in 450 μl of ChIP lysis buffer [50 mm Tris–HCl (pH 8.0), 1% SDS, 10 mm EDTA, 1X cOmplete EDTA-free Protease Inhibitor Cocktail (PIC; Roche)], and lysed on ice for 30 min. Cells were homogenized by six passages through a 20-gauge needle, and nuclei pellets were obtained by centrifugation at 5000 rpm for 10 min at 4 °C. The pellet was resuspended in 900 μl of shearing buffer [10 mm Tris–HCl (pH 8.0), 0.1% SDS, 1 mm EDTA, 1X EDTA-free PIC] and transferred to a 1-ml milliTUBE with AFA fiber (D-Mark Biosciences, Scarborough, ON, Canada) using a 30-gauge needle. Chromatin was sonicated using a Covaris S2 sonicator using the following settings: 10% duty cycle, 6 intensity, 500 burst, 16 cycles (20 s on, 40 s off) at 4–6 °C. Insoluble debris and unfragmented chromatin were removed by centrifugation at 14 000 rpm for 12 min at 4 °C. An aliquot of chromatin was de-crosslinked overnight at 68 °C (0.2 m NaCl, 0.05 mg/ml RNase), treated with proteinase K for 30 min at 42 °C, and purified using the MinElute PCR Purification Kit (28006; Qiagen, Germantown, MD, USA). Concentration of the purified chromatin was determined using the Qubit dsDNA high sensitivity assay (Life Technologies, Carlsbad, CA, USA) and the presence of DNA fragments in the 150–300 bp size range was confirmed by separation on a 2% agarose gel. Protein A Dynabeads (Life Technologies) blocked with bovine serum albumin and salmon sperm DNA for 3 h were used to pre-clear chromatin for 2 h. For immunoprecipitation, three volumes of IP buffer [10 mm Tris-HCl (pH 8.0), 1% Triton X100, 0.1% deoxycholate, 0.1% SDS, 90 mm NaCl, 2 mm EDTA, 1X EDTA-free PIC] were added to 22.5 μg of chromatin, which was then incubated with 5 μg of anti-CIC antibody (Sigma, HPA044341) for 1 h at 4 °C. Blocked Protein A Dynabeads (25 μl) were then added to the chromatin and antibody, and incubated overnight at 4 °C. The samples were then washed twice in low salt buffer [20 mm Tris–HCl (pH 8.0), 0.1% SDS, 1% Triton X100, 2 mm EDTA, 150 mm NaCl] and twice in high salt buffer (same as low salt buffer except 300 mm NaCl). DNA was eluted in 100 mm sodium bicarbonate with 1% SDS and 0.05 mg/ml RNase at 68 °C for 6 h or overnight, followed by treatment with proteinase K for 30 min at 42 °C. For each IP, 20 μl of chromatin was subjected to the same treatment to serve as input controls. Eluted DNA was purified using the MinElute PCR Purification Kit.

Sequencing libraries were prepared following a modified paired-end library protocol (Illumina). In brief, the DNA was subjected to end-repair and phosphorylation using T4 DNA polymerase with Klenow DNA polymerase and T4 polynucleotide kinase, respectively, in a single reaction. 3'-A overhangs were generated using Klenow fragment (3' to 5' exo minus) and ligated to Illumina PE adapters containing 5'-T overhangs. The adapter-ligated products were purified using PCR Clean DX beads (Aline Biosciences), then PCR-amplified with Phusion DNA Polymerase in 13 cycles using Illumina’s PE primer set (Illumina). PCR product was purified using PCR Clean DX beads (Aline Biosciences), and the DNA quality was assessed and quantified using the Caliper LabChip GX DNA High Sensitivity assay (PerkinElmer, American Fork, UT, USA) and the Quant-iT dsDNA high sensitivity assay (Thermo Fisher Scientific). Libraries were normalized and pooled, and the final concentration of the pooled library was determined using a Qubit dsDNA HS Assay Kit and a Qubit fluorometer (Thermo Fisher Scientific). Clusters were generated on the Illumina cluster station and sequence data were generated using an Illumina HiSeq2500 platform following the manufacturer’s instructions.

## **CIC ChIP-seq** **analysis**

BigWig files containing normalized reads [read per kilobase per million mapped reads (RPKM)] in 10 bp bins were generated using Deeptools [50] v.3.0.1. RPKM values within genome-wide 200 bp bins (obtained using Bedtools [51]) were obtained using Deeptools and used to calculate pairwise Spearman correlation values across all libraries. Regions in which CIC ChIP signal was enriched relative to the matched input (i.e*.* peaks) were identified using MACS2 [52] v.2.1.1 with a *q*-value threshold of 0.05.

Published ChIP-seq data derived from *CIC*-WT and *CIC*-KO MEKi-treated (24 h) G144 cell lines [2] were obtained from the ArrayExpress databaseunder the accession number E-MTAB-6682. The obtained data were processed in a manner consistent with our CIC ChIP-seq data, as described above. The matched *CIC*-KO ChIP-seq library was used as the background control for peak calling. Peaks that overlapped between our CIC ChIP-seq and the published set were identified using Bedtools (overlap ≥ 1 bp). The 150 most significant peaks in our dataset were deemed high-confidence CIC peaks based on the inflection point at which the presence of reproducibly identified peaks increased as a function of peak rank relative to all peaks (supplementary material, Figure S2).

Information associated with high-confidence CIC peaks, such as the genomic feature with which they overlapped, the nearest gene, and the peak distance from transcriptional start sites (TSSs), was obtained using ChIPseeker [24]. *De novo* motif enrichment analysis was conducted on high-confidence CIC peaks centred on their summits (defined as the coordinate at which fold-enrichment of ChIP read coverage relative to its matched control was greatest) using HOMER [25] v.4.9.1, with the size parameter set to 200 bp as recommended for identifying primary and co-enriched motifs for TFs (<http://homer.ucsd.edu/homer/ngs/peakMotifs.html>).

## **Histone modification ChIP, library construction, and sequencing**

Samples for native chromatin immunoprecipitation (N-ChIP) were prepared from ~100 000 cells per immunoprecipitation, two replicates per cell line. In brief, cells were lysed using lysis buffer (0.1% Triton X-100, 0.1% deoxycholate) and protease inhibitor for 20 min on ice. The extracted chromatin was then digested using 90 U of MNase enzyme (NEB) for 6 min at 25 °C. Reactions were quenched by adding 5.5 µl of 250 µm EDTA. A mix of 1% Triton X-100 and 1% deoxycholate was then added to the digested samples and the 96-well plate of samples was chilled on ice for 20 min. The digested chromatin was then pooled and 12 µl of chromatin was reserved for use as input control. The rest of the digested chromatin was pre-cleared using IP buffer [20 mm Tris–HCl (pH 7.5), 2 mm EDTA, 150 mm NaCl, 0.1% Triton X-100, 0.1% deoxycholate] plus protease inhibitor with 20 µl of pre-washed Protein A/G Dynabeads (Invitrogen) at 4 °C for 1.5 h. Supernatants were removed from the beads and transferred to a 96-well plate containing the antibody–bead complex. The plate was sealed and incubated at 4 °C on a rotating platform overnight. The reaction plate containing the immunoprecipitation samples was placed on a magnetic plate and samples were washed twice with low salt buffer [20 mm Tris–HCl (pH 8.0), 0.1% SDS, 1% Triton X-100, 2 mm EDTA, 150 mm NaCl] and twice with high salt buffer [20 mm Tris–HCl (pH 8.0), 0.1% SDS, 1% Triton X-100, 2 mm EDTA, 500 mm NaCl]. DNA–antibody complexes were eluted in 30 µl of Elution Buffer (100 mm NaHCO_3_, 1% SDS) and incubated at 65 °C for 1.5 h, with mixing at 1350 rpm on a thermomixer. Protein was digested by adding 1.75 µl of Qiagen Protease to the eluted DNA samples at 50 °C for 30 min, with mixing at 600 rpm on a thermomixer. ChIP DNA was then purified using Sera-Mag beads (Thermo Fisher Scientific) with 30% PEG before library construction. Library construction and sequencing were done identically to the CIC ChIP-seq samples, with the only difference being that the adaptor-ligated products were amplified using eight to ten PCR cycles.

## **Histone modification ChIP-seq analysis**

Duplicated reads were removed using Picard v.1.114 prior to peak calling. FindER (<https://thisisepigenetics.ca/for-scientists/software> [accessed March 2020]) v1.0.1e was used with default parameters to identify peaks. FindER was used due to its ability to accommodate histone modifications with different profile types (localized, broad or a mixture of both). Peaks that overlapped blacklisted regions [53] were first removed. Peaks consistently observed in both replicates were identified and merged using Bedtools to generate the final peak list for subsequent analyses.

For differential enrichment analysis, the union of all peaks across all cell lines was first obtained using Bedtools for each mark. Raw read counts within these regions were obtained using Deeptools. DESeq2 was used to identify differentially enriched (DER) peaks between *CIC*-KO and *CIC*-WT cells (CIC-associated) or between cells expressing IDH1-R132H and cells expressing IDH1-WT cells (IDH1-associated), in a manner analogous to the identification of differentially expressed genes. DER peaks were required to meet a *q*-value threshold of 0.05 and a fold-change of at least 2 to be considered significant, and additionally required directional concordance between both *CIC*-KO replicate cell lines for CIC-associated DER peaks. DER peaks were annotated with their associated genomic feature and nearest gene using ChIPseeker.

As described previously [54], putative enhancer regions were first identified by filtering H3K4me1 peaks that were at least 450 bp long and merging those within 600 bp of each other. H3K4me1 regions within 2 kb of a known TSS were excluded on the basis that these likely corresponded to promoter regions, rather than enhancers. Enhancers overlapping with H3K9me3 peaks were considered to be heterochromatic regions and disregarded in this analysis. Enhancers were assessed for an overlap with DER H3K4me1, H3K27ac, and H3K27me3 peaks using Bedtools and were considered candidate dysregulated enhancers or differentially enriched enhancers (DER enhancers) if an overlap was present with at least one of these marks. The nearest genes associated with DER enhancers were considered to be putative targets of such enhancers. *De novo* motif analysis was performed on downregulated and upregulated DER enhancers using HOMER, with the size parameter set to 500 bp as recommended for histone marked regions (http://homer.ucsd.edu/homer/ngs/peakMotifs.html) and otherwise default parameters.

## **Whole genome bisulphite sequencing and data processing**

Whole genome bisulphite sequencing was performed on two replicates per cell line. To track the efficiency of bisulphite conversion, 10 ng of lambda DNA (Promega, Madison, WI, USA) was spiked into 1 µg of genomic DNA, quantified using Qubit fluorometry, and arrayed in a 96-well microtitre plate. DNA was sheared to a target size of 300 bp using Covaris sonication [2 × 50 s at a ‘Duty cycle’ of 30%, 450 Peak Incident Power (W), and 200 Cycles per Burst] and the fragments were subjected to end-repair and phosphorylation in a single reaction, using an enzyme premix (NEB) containing T4 DNA polymerase, Klenow DNA polymerase, and T4 polynucleotide kinase, and incubated at 20 °C for 30 min. Repaired DNA was purified in 96-well format using PCR Clean DX beads (Aline Biosciences), and 3'-A-tailed (adenylation) using Klenow fragment (3' to 5' exo minus) at 37 °C for 30 min prior to enzyme heat inactivation. Cytosine methylated paired-end adapters (5'-mCAmCTmCTTTmCmCmCTAmCAmCGAmCGmCTmCTTmCmCGATmCT-3' and 3'-GAGmCmCGTAAGGAmCGAmCTTGGmCGAGAAGGmCTAG-5') were ligated to the DNA at 20 °C for 15 min and adapter flanked DNA fragments bead-purified. Bisulphite conversion of the methylated adapter-ligated DNA fragments was achieved using the EZ Methylation-Gold kit (Zymo Research, Irvine, CA, USA), following the manufacturer’s protocol. Five cycles of PCR using HiFi polymerase (Kapa Biosystems, Wilmington, MA, USA) was used to enrich the bisulphite-converted DNA. Post-PCR purification and size selection of bisulphite-converted DNA were performed using 1:1 PCR Clean DX beads. To determine final library concentrations, fragment sizes were assessed using the DNA 1000 assay (Agilent) and DNA was quantified using Qubit fluorometry. Clusters were generated on the Illumina cluster station and sequence data were collected on the Illumina HiSeq X platform following the manufacturer’s instructions. Sequencing reads were aligned to the human reference genome GRCh37-lite using NovoAlign (http://www.novocraft.com/products/novoalign). Fractional methylation values were obtained for each aligned CpG with a minimum coverage of 5 reads using NovoMethyl (http://[www.novocraft.com](http://www.novocraft.com) [accessed March 2020]).

## **Differential methylation analysis**

Differentially methylated regions (DMRs) were identified using Defiant [26] with default parameters (minimum CpG coverage of 5, *P* value cut-off of 0.05, minimum methylation difference = 10%, and minimum number of CpGs in a DMR = 5). DMRs between replicate *CIC*-KO cell lines were assessed for both overlap (≥1 bp) and concordant directionality, and were considered to be CIC-associated if they met these criteria. Bedtools was used to overlap DMRs with genomic features and ChIPSeeker was used to identify their nearest genes. Calculation of average fractional methylation of genomic regions (e.g. CIC peaks) was also performed using Bedtools.
