# Supplementary material for: Integrative multi‐omic analysis reveals neurodevelopmental gene dysregulation in CIC ‐knockout and IDH1 ‐mutant cells
Source: J Pathol. 2021 Dec 22;256(3):297–309. doi: 10.1002/path.5835 (PMC9305137; doi:10.1002/path.5835)
Supplement: Supplementary file 2 — Figure S1. Confirmation of CIC and IDH1 status in cell line models Figure S2. Number of reproducibly identified CIC peaks versus MACS2 q‐value significance Figure S3. Known CIC target genes are overexpressed in CIC‐KO cells Figure S4. Comparison of peaks across all cell lines for each histone modification Figure S5. Summary of DER peaks Figure S6. CIC binding is not associated with differential methylation Figure S7. CIC‐associated differential methylation is not associated with differential gene expression [file PATH-256-297-s001.docx]

**Integrative multi-omic analysis reveals neurodevelopmental gene dysregulation in *CIC*-knockout and *IDH1*-mutant cells**

SD Lee *et al. J Pathol* DOI: 10.1002/path.5835

**Supplementary Figures S1–S7**

Reference numbers refer to the main text list


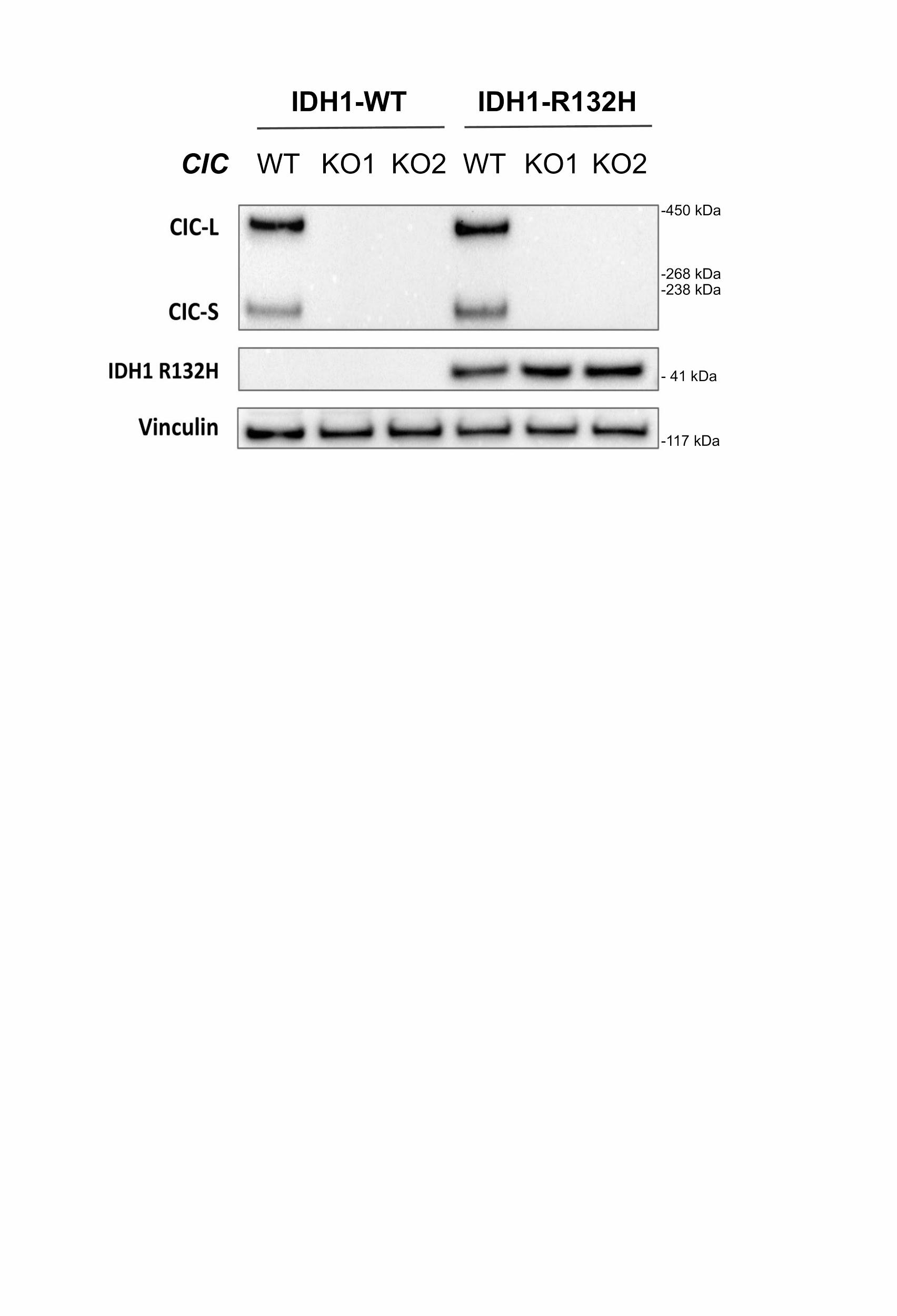


**Figure S1. Confirmation of CIC and IDH1 status in cell line models.** Western blots for CIC, IDH1-R132H, and vinculin confirming the absence of CIC protein in the *CIC*-KO cell lines and the presence of IDH1-R132H protein in the *CIC*-WT (IDH1-R132H) line and its progeny. Western blots were performed on whole-cell lysates harvested from the same cell line thaw as those submitted for transcriptomic and epigenomic profiling.


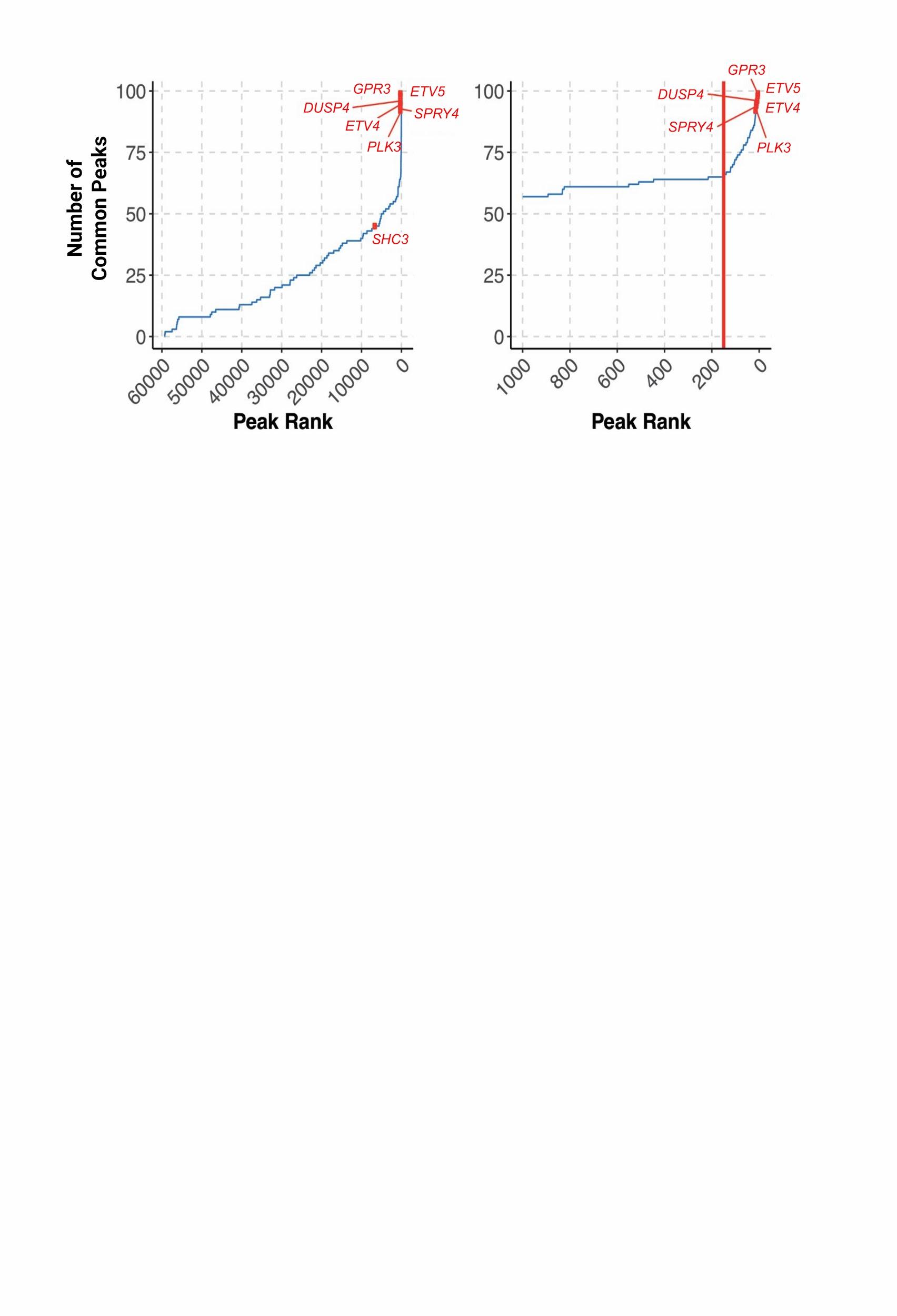


**Figure S2**. **Number of reproducibly identified CIC peaks versus MACS2 *q*-value significance.** CIC peaks ranked by significance (MACS2 *q*-value) versus the number of peaks in common with a published CIC ChIP-seq dataset [2] (Materials and methods). The left plot shows all peaks, while the right plot shows a subset of the top 1000 most significant peaks. The genes for which a CIC peak was detected in the vicinity of their TSS and were previously validated by ChIP-qPCR are shown in red. The point of inflection at which the number of reproducibly identified peaks increased relative to the entire curve was identified to be approximately at the 150th ranked peak (red line).


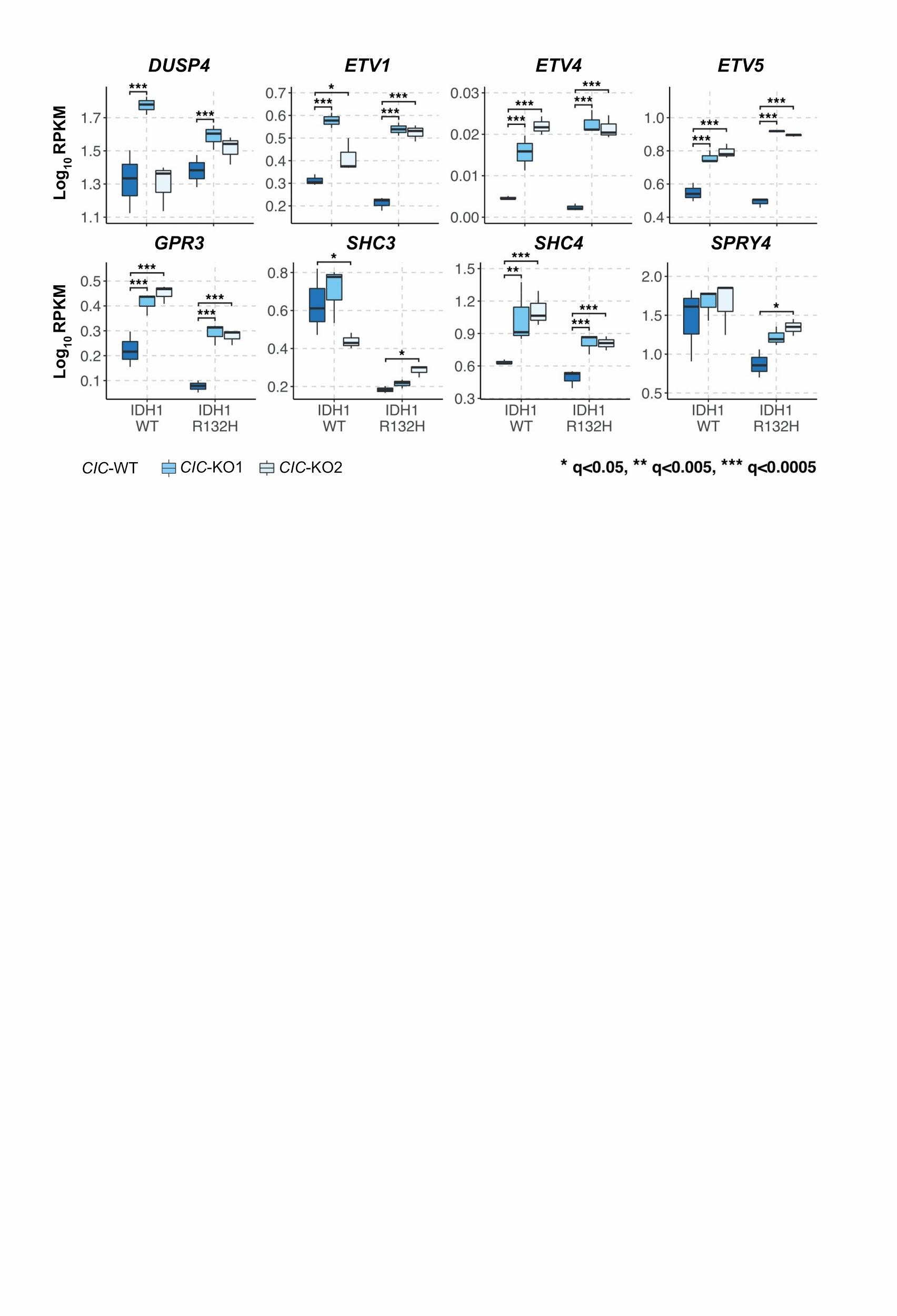


**Figure S3**. **Known CIC target genes are overexpressed in *CIC*-KO cells.** Expression levels (RPKM) of known CIC target genes in each cell line relative to the *CIC*-WT (IDH1-WT) parental cell line. The number of stars (legend at bottom right) corresponds to the *q*-value obtained from DESeq2.


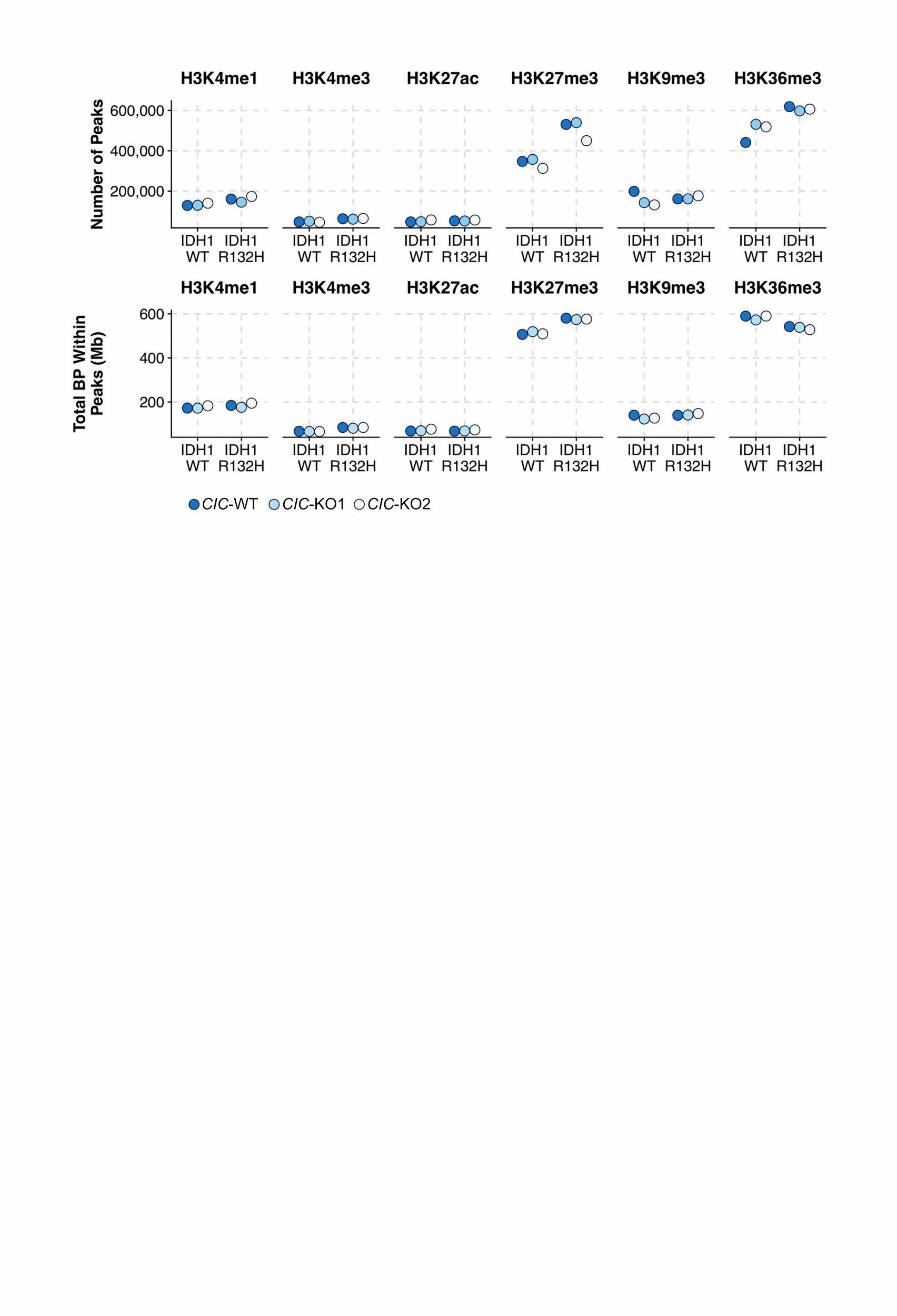


**Figure S4. Comparison of peaks across all cell lines for each histone modification.** The mean number of peaks identified using FindER (*q* < 0.05) and base pairs within peaks (in megabases) across replicate pairs for each histone modification library.


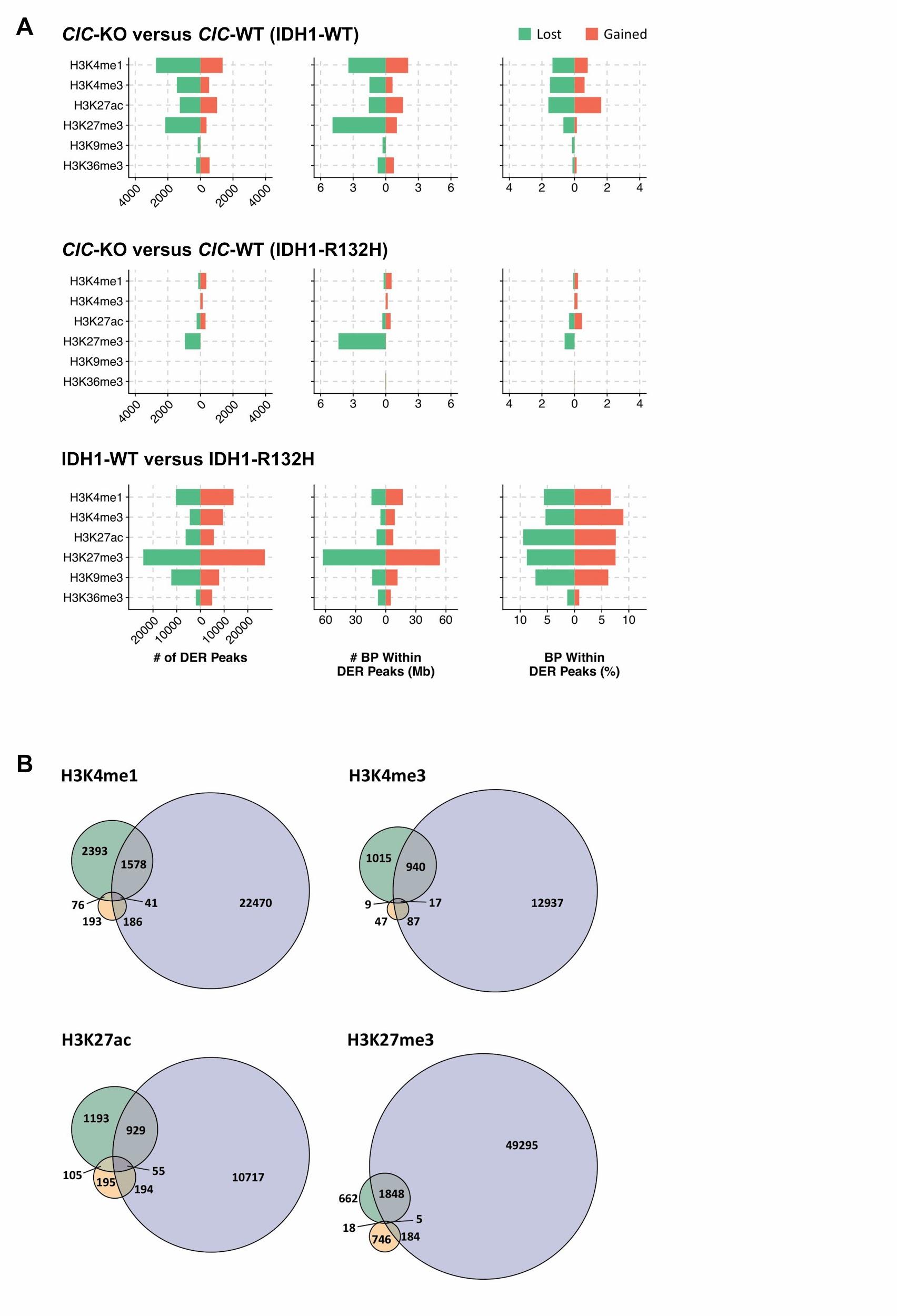


**Figure S5.** **Summary of DER peaks.** (A) Summary of significant DER peaks (*q* < 0.05, fold-change > 2) across all histone modifications and for all three comparisons. Metrics shown are as follows: number of DER peaks (left), number of base pairs covered by DER peaks (centre), and base pairs within DER peaks as a percentage of total base pairs within peaks for each mark (right). Bars are coloured according to the direction of differential enrichment (loss = green; gain = red). (B) Venn diagrams displaying the intersections of all DER peak analyses for H3K4me1, H3K4me3, H3K27ac, and H3K27me3. CIC-associated DER peaks comprised those that were concordantly DER between replicate *CIC*-KO cell lines.


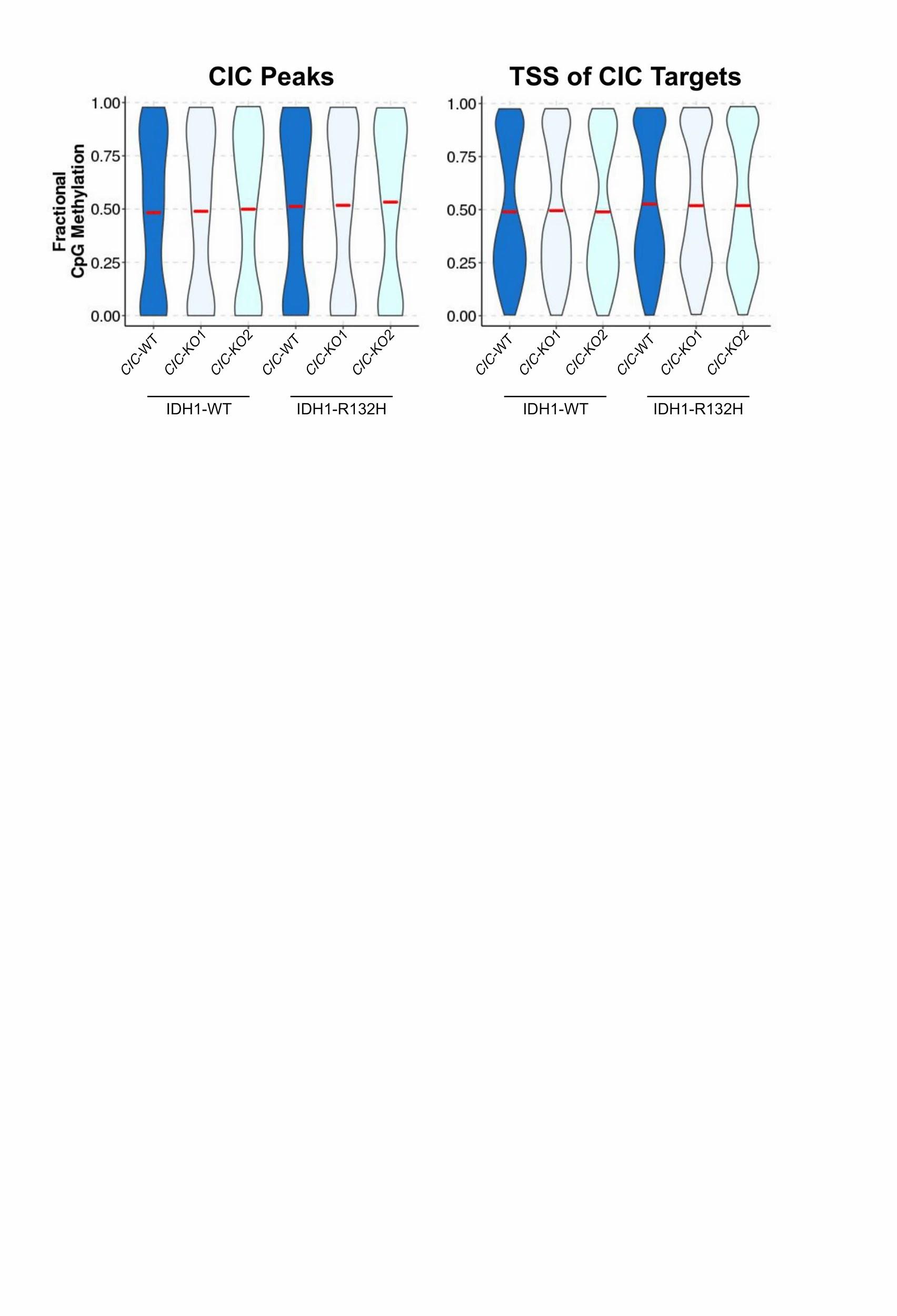


**Figure S6.** **CIC binding is not associated with differential methylation.** Violin plots of fractional CpG methylation of CIC peak regions (left) and at ± 2 kb around the TSSs of putative CIC targets (right) across all cell lines. Very little difference in the distributions comparing *CIC*-KO cells with their WT counterparts is evident. Red cross bars represent the means.


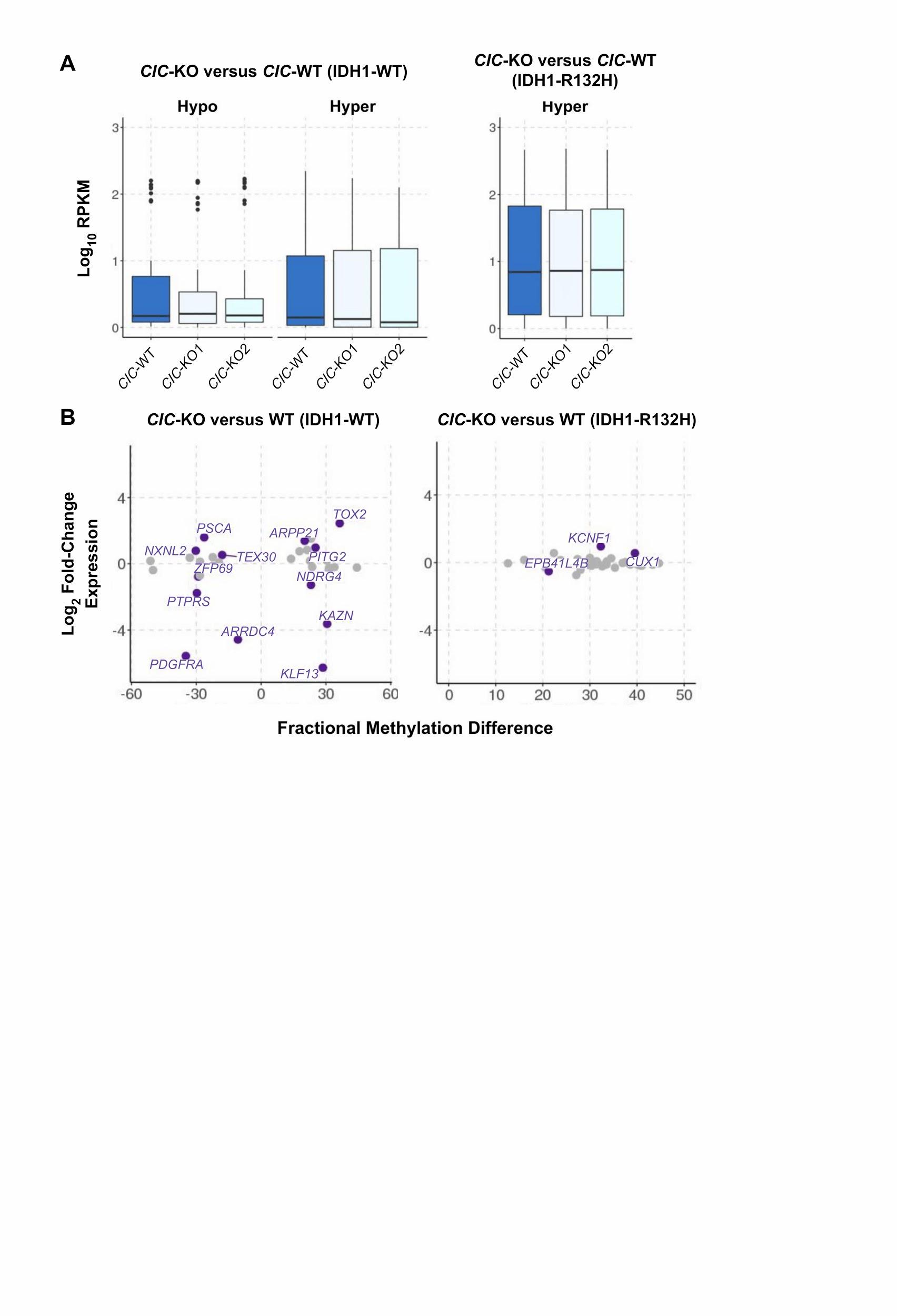


**Figure S7.** **CIC-associated differential methylation is not associated with differential gene expression**. (A) Expression levels (RPKM) of genes associated with CIC-associated hypo- and hyper-methylated promoter CpG islands across *CIC*-WT and *CIC*-KO cell lines. Since no CIC-associated hypomethylated DMRs were found at promoters in the IDH1-R132H cell lines, only the expression of genes associated with hypermethylated DMRs is shown. (B) Fractional methylation differences of promoter CpG island DMRs (*x*-axis) and changes in expression (log_2_ fold-change RPKM, *CIC*-KO versus WT) of downstream genes *(y*-axis). Non-significant DE genes are coloured grey, while significant DE genes are coloured purple and labelled.
